# Predicting obsessive-compulsive disorder episodes in adolescents using a wearable biosensor—A wrist angel feasibility study

**DOI:** 10.3389/fpsyt.2023.1231024

**Published:** 2023-10-02

**Authors:** Nicole Nadine Lønfeldt, Kristoffer Vinther Olesen, Sneha Das, Anna-Rosa Cecilie Mora-Jensen, Anne Katrine Pagsberg, Line Katrine Harder Clemmensen

**Affiliations:** ^1^Child and Adolescent Mental Health Center, Copenhagen University Hospital—Mental Health Services Copenhagen (CPH), Hellerup, Denmark; ^2^Department of Applied Mathematics and Computer Science, Technical University of Denmark, Kgs Lyngby, Denmark; ^3^Department of Clinical Medicine, Faculty of Health and Medical Sciences, University of Copenhagen, Copenhagen, Denmark

**Keywords:** obsessive-compulsive disorder, children, adolescents, machine learning, signal processing, wearable biosensor

## Abstract

**Introduction:**

Obsessive-compulsive disorders (OCD) are marked by distress, negative emotions, mental processes and behaviors that are reflected in physiological signals such as heart rate, electrodermal activity, and skin temperature. Continuous monitoring of physiological signals associated with OCD symptoms may make measures of OCD more objective and facilitate close monitoring of prodromal symptoms, treatment progress and risk of relapse. Thus, we explored the feasibility of capturing OCD events in the real world using an unobtrusive wrist worn biosensor and machine learning models.

**Methods:**

Nine adolescents (ages 10–17 years) with mild to moderate-severe OCD were recruited from child and adolescent mental health services. Participants were asked to wear the biosensor in the lab during conditions of rest and exposure to OCD symptom-triggering stimuli and for up to 8 weeks in their everyday lives and register OCD events. We explored the relationships among physiological data, registered OCD events, age, OCD symptom severity and symptom types. In the machine learning models, we considered detection of OCD events as a binary classification problem. A nested cross-validation strategy with either random 10-folds, leave-one-subject-out, or leave-week(s)-out in both layers was used. We compared the performance of four models: logistic regression, random forest (RF), feedforward neural networks, and mixed-effect random forest (MERF). To explore the ability of the models to detect OCD events in new patients, we assessed the performance of participant-based generalized models. To explore the ability of models to detect OCD events in future, unseen data from the same patients, we compared the performance of temporal generalized models trained on multiple patients with personalized models trained on single patients.

**Results:**

Eight of the nine participants collected biosensor signals totaling 2, 405 h and registered 1, 639 OCD events. Better performance was obtained when generalizing across time compared to across patients. Generalized temporal models trained on multiple patients were found to perform better than personalized models trained on single patients. RF and MERF models outperformed the other models in terms of accuracy in all cross-validation strategies, reaching 70% accuracy in random and participant cross-validation.

**Conclusion:**

Our pilot results suggest that it is possible to detect OCD episodes in the everyday lives of adolescents using physiological signals captured with a wearable biosensor. Large scale studies are needed to train and test models capable of detecting and predicting episodes.

**Clinical trial registration:**

ClinicalTrials.gov: NCT05064527, registered October 1, 2021.

## 1. Introduction

Obsessive-compulsive disorder (OCD) in children and adolescents is a chronic and debilitating psychiatric disorder that can negatively affect school performance, strain family relations and friendships, and shorten life expectancy (1, 2). OCD occurs in up to 3% of children and adolescents under the age of 18 years (3). The first defining symptom of OCD, obsessions, are intrusive, persistent thoughts about topics such as contamination, symmetry, morality and committing aggressive acts (4). The second characteristic symptom of OCD, compulsions, refers to ritualized or repetitive acts that the individual with OCD feels compelled to do, such as checking, cleaning, ordering, counting and hoarding (4). Obsessions and compulsions are associated with distress in the form of negative emotions (e.g., anxiety, fear, disgust, sadness, embarrassment, feelings of incompleteness, and anger) (4) and cognitive effort (associated with monitoring, uncertainty and suppression) (5).

Assessing distress levels in OCD is important for determining symptom severity (6), which informs treatment choice and planning. Monitoring of OCD distress is critical to delivering and studying the first line treatment for OCD, cognitive behavior therapy (CBT) with exposure and response prevention (ERP) (7). ERP involves approaching distress provoking stimuli (exposure) and refraining from performing rituals or safety behaviors (response prevention). Currently, monitoring OCD distress occurs via self-report and is not continuous but intermittent. Actual continuous, automatic, objective measures of distress would enable more frequent and precise observations and, in turn, just-in-time interventions.

The distress, negative emotions, and cognitive effort evoked by OCD are reflected in autonomic nervous system activity (e.g., heart rate, electrodermal activity) (8, 9). Biofeedback of physiological markers may have a direct therapeutic effect. For example, one study found that providing heart rate feedback to 54 university students (mean age nearly 18 years) with symptoms of claustrophobia during exposure sessions showed significantly more habituation between exposure sessions and reduced fear at the end of six 5-min exposures compared to controls who did not receive heart-rate feedback (instead heard paced tone or nothing) (10).

Wearable biosensors are available that can monitor autonomic nervous system activity, but research on the application of these biosensors for continuous monitoring within mental health care is still in its infancy. Most studies have focused on stress and depression in adults. Using measures of autonomic nervous system activity from a wrist worn biosensor (Empatica's E4) as input, machine learning models classified no stress, low stress and high stress under controlled stress or no stress conditions (e.g., a stressful timed and evaluated arithmetic task) with 72% accuracy (11). Another study used physiological stress information (measured with the E4) from lab experiments and contextual information to detect stress-events (with a minimum duration of 1 h) every 20 min with 92% accuracy (11). An early study demonstrated lower heart rate variability (measured with biosensors built into a vest) in adults with OCD, panic, social, and generalized anxiety disorders than healthy controls (12). One study focused on OCD symptoms in a convenience sample of 21 adults and found that machine learning models could correctly distinguish enacted compulsive hand washing from routine hand washing with a sensitivity rate of 84% and a specificity rate of 30% (13).

Fewer studies have applied wearable biosensors to the field of child psychiatry. Here, the focus has been on children with autism, to a lesser extent, attention deficit hyperactivity disorder, and one study examined internalizing disorders (14). More than half of these studies employed biosensors worn on the chest or multiple sites on the body. The sample sizes of most studies were quite small, with an average of 27 participants (14). One study was able to classify emotion dysregulation in five 8-12-year-old children using signals from the E4 with 68–85% accuracy (15). Another study of children and adolescents (ages 6–17 years; *N* = 20) with autism, predicted aggressive events 1 min before the episode using 3 min of physiological and motion signals measured with the E4 (area under the curve: 0.71–0.84) (16). We know of only one study that has tested the use of biosensors to detect OCD events in youth. A pilot study placed a chest belt with an electrocardiograph, inertial sensors worn on both wrists and a head mounted eye tracker on five adolescents (ages 13–17 years) with OCD while performing regular daily activities, exposure preparation, exposure, and compulsions (17). No machine learning models were trained or tested to classify OCD events. However, the study provides a protocol and preliminary support for distinct physiological stress patterns (increased HR and decreased heart rate variability) in exposure situations (17).

To our knowledge, no previous study has attempted to detect OCD events outside of controlled conditions. Thus, we tested the feasibility of capturing OCD events using an unobtrusive wrist worn biosensor, E4, and machine learning models in a sample of adolescents with an OCD diagnosis. To succeed in continuous monitoring of OCD symptoms, adolescents would need to adhere to wearing the E4 and tag enough OCD events to amass enough data for analysis. We characterized the biosensor wearing behavior per day and over the entire observation period, as well as explored the relationships between OCD symptoms, wearing behavior and how often the adolescents tagged OCD events. We used these observations to generate hypotheses about OCD symptoms that can be monitored using physiological signals and the characteristics of adolescents with OCD who would benefit from OCD symptom monitoring with a biosensor. We also took a preliminary step toward developing and testing generalized and personal models for detecting OCD events in the everyday lives of adolescents. Finally, we compared the physiological signals collected outside of the lab to those collected during OCD-activating tasks in the lab.

## 2. Materials and methods

This study was approved by the Ethics Committee of the Captial Region of Denmark on June 17, 2021 (ref. nr. H-18010607-79689). A detailed description of the design of this prospective study was reported in the protocol (18). Here, we focus on physiological data collected *in-the-wild* and at Time (T) 1 in the clinic.

### 2.1. Participants

Participants were recruited from the child and adolescent mental health center within the Capital Region of Denmark hospital system. Inclusion criteria were primary or secondary diagnosis of OCD, OCD severity score of seven or above [Children's Yale-Brown Obsessive Compulsive Scale (CY-BOCS) (19)], receiving care or on wait list for care within the mental health center, normal intellectual function, ages 8–17 years and signed informed consent from the legal guardian. Exclusion criteria were substance dependence, schizophrenia, psychotic disorders, mania, bipolar disorder, pervasive developmental disorder (with the exception of Asperger syndrome), current participation in other OCD trials, or any condition that would hinder wearing a biosensor on the wrist. Nine adolescents (five females and four males) between the ages of 10 and 16 years (mean age in years = 12.3, SD = 2.6) who were diagnosed with OCD [F42.2 according to the International Statistical Classification of Diseases and Related Health Problems (ICD-10) (20)] participated in this study. At enrollment, participants' OCD severity scores (CY-BOCS total score), ranged from 11 (mild) to 29 (moderate-severe; mean = 24.56, SD = 5.12). Four of the participants had previously received some form of psychosocial treatment for OCD. One participant had received antipsychotic medication prior to the observation period. During the observation period two participants were receiving psychosocial treatment and one was receiving sertraline for OCD. Two participants also had another diagnosis Asperger syndrome [F84.5 (20)] and asthma. The adolescents were of normal intelligence as reported by parents or the age-appropriate intelligence test (IQ range = 95–113) (21, 22).

### 2.2. Measures

#### 2.2.1. Clinical

OCD symptom severity was assessed with the gold-standard measure, the CY-BOCS (19). Each CY-BOCS interview was conducted by one of two investigators in this study: a trained medical doctor and a psychologist. Five items are summed to obtain a severity score for obsessions and another five items are summed to obtain a severity score of compulsions. When all ten of these items are summed, it yields a total OCD severity score that can range from 0 to 40 (19). We also summed pairs of obsession and compulsion severity items for time occupied by symptoms (items 1a and 6a), interference caused by symptoms (items 2 and 7), distress caused by symptoms (items 3 and 8), resistance against symptoms (items 4 and 9), and control over symptoms (items 5 and 10). OCD symptom types and counts of symptoms were determined using the checklist of the CY-BOCS. We summed the number of symptoms endorsed within the following categories that have been supported by factor analysis: symmetry, forbidden thoughts, cleaning, and hoarding (23, 24). All endorsed symptoms were summed to obtain a total symptom count.

#### 2.2.2. Physiological

Participants were asked to wear an E4 (25) on their nondominant hand. The E4 wristband measures blood volume pulse (BVP; sampling rate: 64 Hz), external skin temperature (sampling rate: 4 Hz), and electrodermal activity (EDA; sampling rate: 4 Hz). Heart rate (HR; sampling rate: 1 Hz) was calculated using the BVP signal. The E4 also includes a button to mark events.

### 2.3. Procedure

Participants were asked to wear a biosensor everyday during their daily routines from the time they awoke until they went to bed for eight weeks. We asked participants to press the event mark button on the biosensor whenever they were bothered or stressed by their OCD symptoms. To protect the privacy of the participants, the data was never connected to participant mobile phones. Instead, data was downloaded from the E4 wristband to the E4 server via a USB cable during in-person meetings that occurred up to twice per week and then transferred to the hospital server. At these meetings participants had the opportunity to report problems they experienced with the biosensor, instances of and reasons for not wearing the biosensor and if they falsely tagged any events. Any reports were recorded in the data capturing system, RedCap (26). Before (T1) and after (T2) the 8-week observation period, participants were assessed for OCD symptoms and severity. At T1 and T2 participants also wore the biosensors under controlled conditions: a 5-min resting period, in which they were asked to sit quietly in a room with their parent while the investigator(s) waited outside and an ERP session, in which participants approached OCD symptom inducing stimuli and refrained from rituals and other safety behaviors. The ERP session had an preparation phase, an exposure phase, and a debriefing phase. The ERP sessions were video and audio recorded and timestamps were placed on the start and stop times of exposure. OCD symptom categories, severity ratings and experiments from T1 are reported in this study.

### 2.4. Data analyses

The analysis plan for this study was registered prior to data analysis (27). We performed analyses designed to explore the acceptability of the biosensor, generate hypotheses about the associations between biosensor use and OCD symptom characteristics, and explore the performance of models using features from a biosensor to detect OCD-distress episodes. We evaluated acceptability by examining adherence to wearing the biosensor for the entire study including days when data was missing due to technical or logistical problems involving the biosensor. We summed the number of hours and days participants wore the biosensor and the number of tagged events. We also explored participant use of the biosensor by hour, hours per day and per week by calculating the mean number of hours of physiological data recorded per day and the mean number of tagged events per hour for each participant. To explore the relationships among participant characteristics and biosensor data, we calculated Spearman rank-correlations between number and type of OCD symptoms, symptom severity (subscales and total), age, amount of recorded data, and number of tagged events (significance threshold: *p*<0.01).

**Figure 1:**
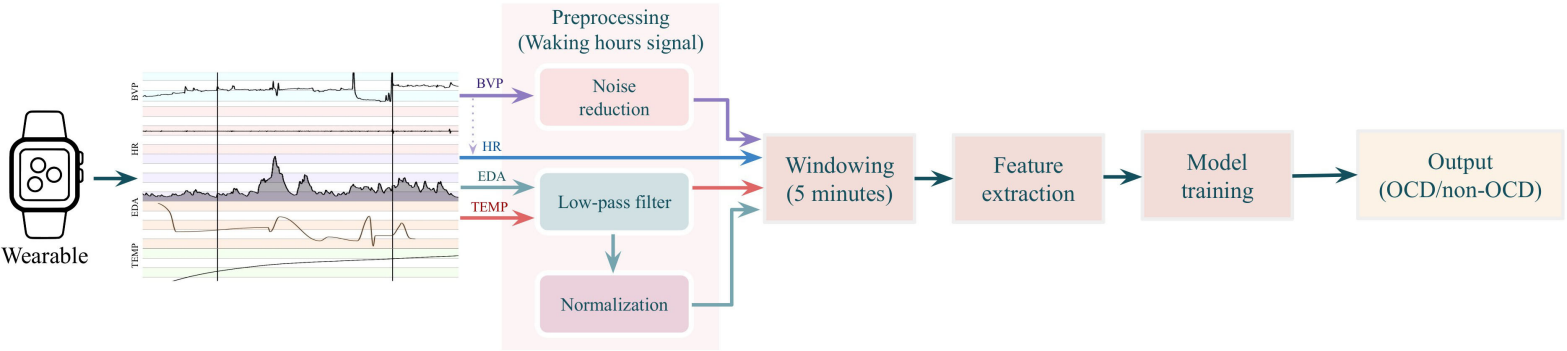
Model development flowchart. Physiological signals—blood volume pulse (BVP), heart rate (HR), electrodermal activity (EDA), and skin temperature (TEMP)—from waking periods were preprocessed and then segmented into 5-min windows. Next, 66 features were extracted and entered into supervised machine learning models, which classify the 5-min segments of physiological data as OCD events or nonevents and are OCD events.

**Figure 2:**
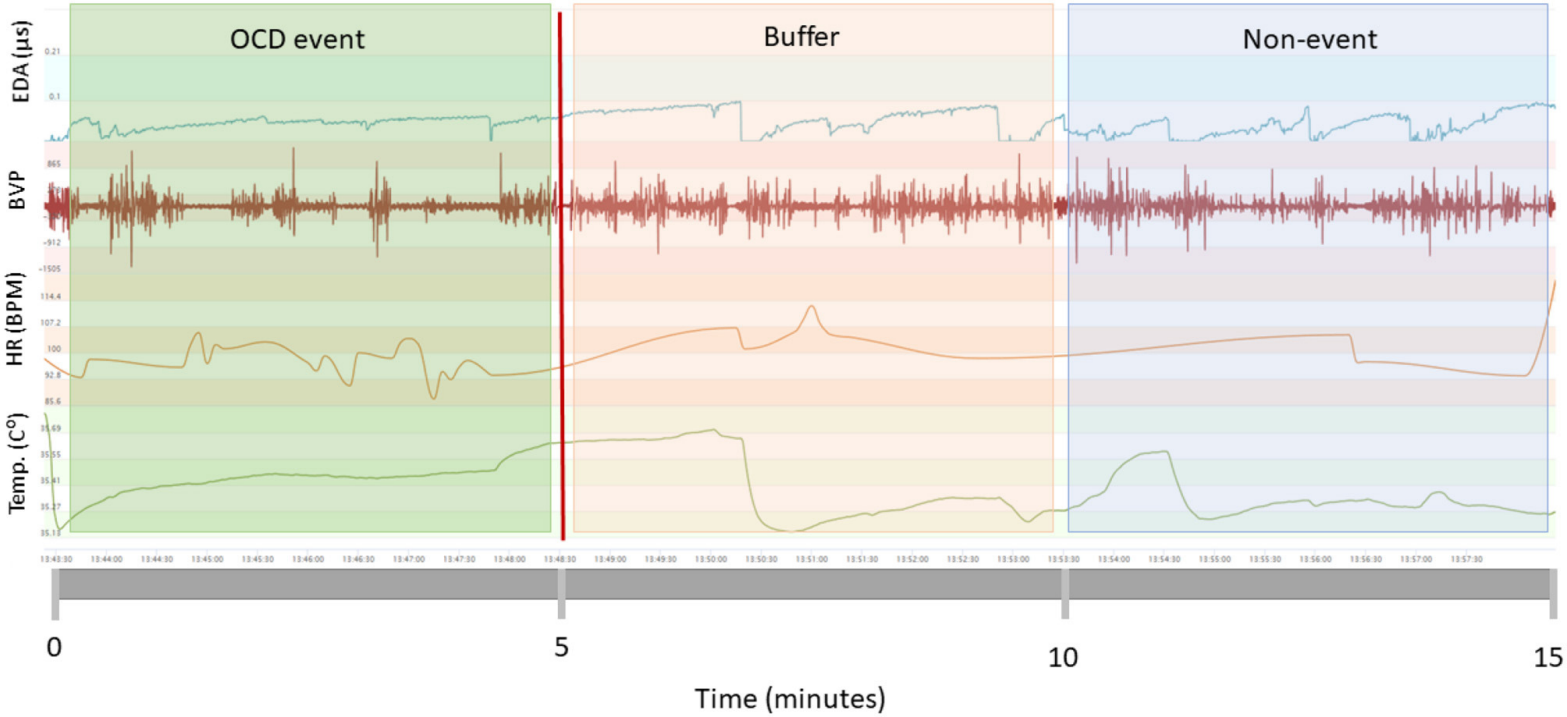
Windowing strategy. OCD events (green box) were defined as the 5-min window prior to patient tags (vertical red line). The 5 min following patient tags were designated as buffer zones (red box) meaning they could not be OCD events nor non-events. Five minute segments following the buffer zones were designated as non-events (blue box).

Figure 1 provides an overview of the steps involved in model development including model inputs, signal preprocessing, feature extraction, model training and classification. Before model training, the biosensor data was filtered to remove periods when the participant was sleeping or when the participant was not wearing the E4. The process of model development is displayed in Figure 1. All features were preprocessed, transformed and calculated for 5-min intervals. In previous studies, interval length for stress and anxiety detection has ranged from 10 s to 4 min (11, 28, 29). Although shorter intervals can reflect changes in mental state (30), a precedence of at least 100 s has been set to allow to autonomic responses to extinguish (31). In this initial study, we expanded the interval to 5 min to hopefully increase the likelihood of capturing OCD events as participants labeled OCD events in real time. We expect that there is a delay between awareness of OCD stress and labeling as found in previous studies (11). Furthermore, OCD event labels were registered as point events. Thus, we have no information on the duration of the events. OCD events were defined as a time window from 5 min prior to the event tag to the event tag (see Figure 2). To ensure no overlap of OCD events and nonevents, we created a buffer period by removing 5 min of data (tags and physiological signals) after each event tag. Nonevent windows of 5 min were randomly sampled from the remaining time periods. We sampled a minimum of three nonevents from each recording session to ensure data from each day was included.

In total, we extracted 66 features from each 5-min window (see Supplementary Table S3 for full list and descriptions). BVP contributed 24 features; HR contributed 9; 5 came from skin temperature and 28 come from EDA. All 66 features were included in the models. All extracted features were standardized to zero mean and unit variance.

#### 2.4.1. Model training

**Figure 3:**
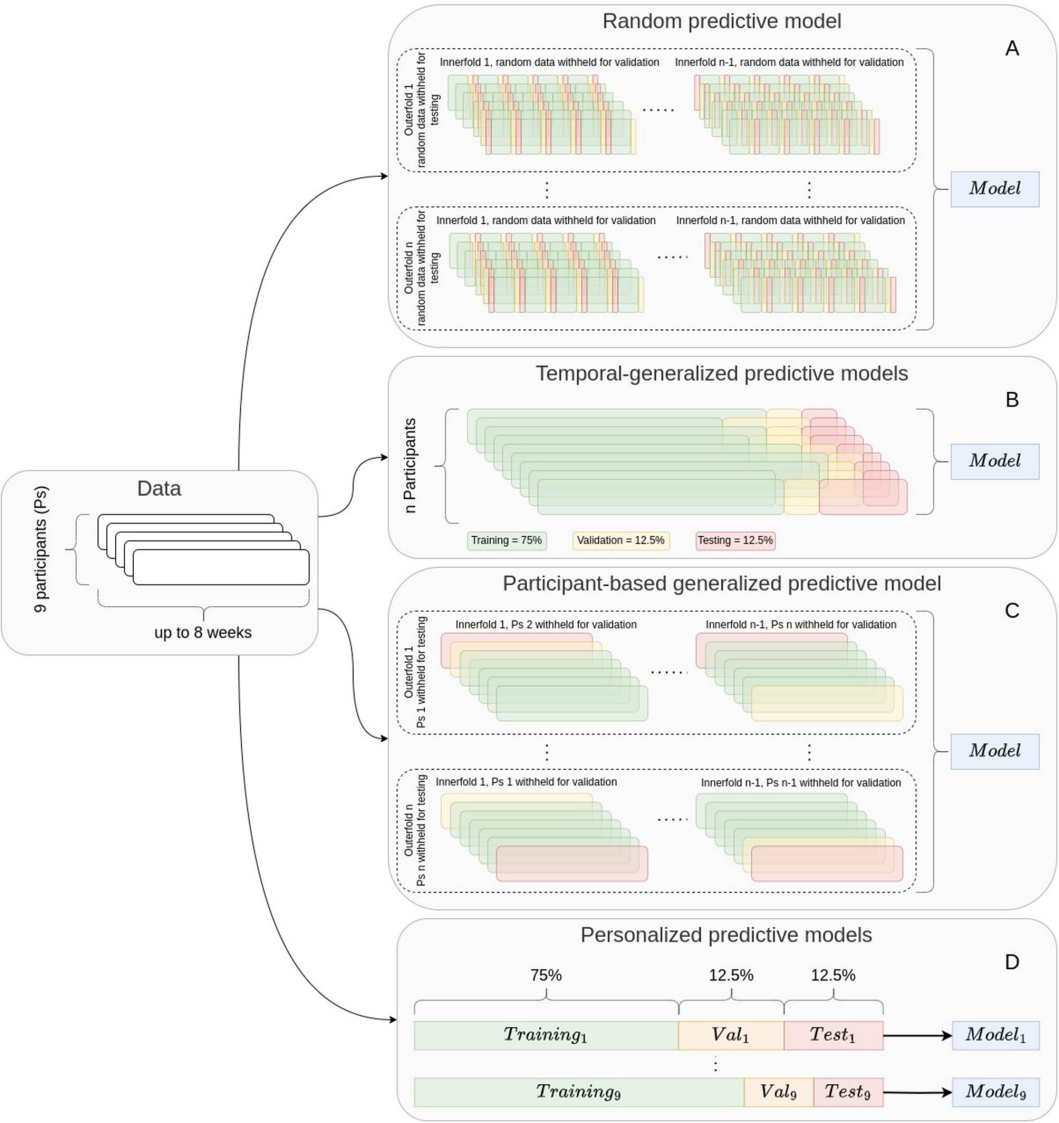
Cross-validation strategies. Models using random cross-validation **(A)**, generalized predictive models **(B, C)** and personalized predictive models **(D)** were trained. Temporal generalized models **(B)** and personalized predictive models **(D)** used a leave-1-week-out strategy. The participant-based generalized predictive models **(C)** used a leave-one-person-out strategy. This figure is a modified version of the figure published in Olesen et al. (27), which was published under the terms of Creative Commons Attribution 4.0 license.

We treated the prediction of OCD events as a binary classification problem, in which each 5-min window was considered an independent observation marking either an OCD event or non-OCD event. Due to the limited amount of data, we employed nested cross-validation with the outer and inner layers being a random 10-fold-cross-validation. Within each fold, the positive observations (OCD events) in the training set were over-sampled to match the number of negative observations (non-OCD events). Nested cross-validation uses the outer layer to evaluate the performance of the models trained on the inner layer and selects the best model and hyperparameters using the inner layer in an iterative process (see more information in the Supplementary Table S4). Using this nested cross-validation method, we evaluated three scenarios representing different clinical applications (Figure 3). Specifically, the data were split into training, validation, and test sets: (1) In the *time-split scenario* (B in Figure 3), the first 75% of observation days for all participants formed the training set, the next 12.5% of the observation period was designated as the validation set and the final 12.5% of the observation period formed the hold-out test set. Model performance was based on 10 repetitions with the same hold-out set. With this evaluation method, we assessed model performance as if it were trained on 5 weeks of all patient data, and then used to detect future OCD events for the same patients. (2) In the *participant-split scenario* (C in Figure 3), the inner layer models were trained on all but two participants, which served as the validation and test sets and outer layer was leave-one-subject-out. With this evaluation method, we assessed model performance as if it were trained on specific patients in the clinic, and then used to detect OCD events in new patients. The time-split and participant-split scenarios were also compared to a *random-split scenario* (A in Figure 3), in which data were randomly split into training (75%), validation (12.5%), and test sets (12.5%). Model performance for random and participant-based cross-validation was evaluated as the average of the testing folds of the outer layer. For participant-based model performance, the average was weighted by the population percentage of each patient.

(3) In the *personalized time-split scenario* (D in Figure 3), a separate model was trained for each individual using the first 75% of the individual's observation period for training and the subsequent two 12.5% sets of the observational period for validation and test sets. With this evaluation method, we assessed model performance as if it were trained on about 5 weeks of data, and then used to detect future OCD events for the same patient. We compared the performance of personalized predictive models with the performance of the temporal generalized models. To explore the differences between model performance for each participant, we calculated the mean increase by subtracting the personalized model performance metrics from the generalized model performance metrics and performed *t*-tests. However, we note that the sample size is too small to draw any conclusions from these tests. These analyses were exploratory to inspire hypotheses to be tested in sufficiently large samples.

In each scenario, the outer layer estimated model performance on the hold-out test set using the following metrics: accuracy, F1-score, precision, recall, and area under the receiver operating characteristic (ROC-AUC). These metrics are defined in Section 7 of the Supplementary material. The inner layer compared the performance of feedforward neural networks (NN), logistic regression (LR), random forest (RF), and mixed-effect random forest (MERF) model and selected the best performing model and hyperparameters based on accuracy. These models were chosen as a starting point to explore the feasibility of detecting OCD events in-the-wild. The size of our sample was not ideal for training generalizable models. Nonetheless, we experimented with a simple deep learning technique, NN. Given our small sample, LR and RF are most suitable as they are less prone to overfitting (32). MERF can account for relationships within clustered, longitudinal data.

#### 2.4.2. Evaluation of feature importance

To examine feature importance and increase interpretability of our models, we plotted SHapley Additive exPlanations (SHAP) values (33) for the random cross-validation models. Positive SHAP values indicate that features have a positive impact on the prediction of a model, i.e., leading the model to classify an observation as an OCD event (33). Negative SHAP values negatively impact prediction, i.e., influencing the model to classify an observation as a nonevent (33).

#### 2.4.3. Features during controlled OCD events and nonevents

We extracted the most important features for differentiating OCD events from nonevents in-the-wild from data collected in the laboratory at T1 under conditions of rest and exposure, in which OCD symptoms were evoked. Using data from 5 min of the resting period and the first 5 min of the exposure period, we created box plots to examine differences in features between conditions of rest and exposure. Five of eight participants had E4 recordings under these controlled conditions. The exposure phase of the start and stop time of the exposure sessions were identified using the video recordings of the sessions. Three participants were removed from this analysis as either their exposure session video was missing or the E4 timestamps did not align with the video timestamps.

#### 2.4.4. Power analysis

To estimate the amount training data needed to achieve a certain level of performance, we fitted the inverse power law to the measured performance metrics as a function of the number of observations (34). The performance metrics are expressed as accuracy, F1-score, precision, recall and ROC-AUC. To remove the uncertainty of between participant variance, we calculated performance levels by using personalized models. Models were run several times: once using 100% of the participant observations and five times using incrementally down-sampled observations repeated ten times. We down-sampled the training data to 10, 25, 50, 75, and 90% through stratified random sampling to maintain the same class (OCD event, nonevent) distribution. Thus, we ran models for participants with many observations (OCD events and nonevents). The test set was held constant in all models.

## 3. Results

### 3.1. Acceptability

Details of the amount of data collected from the biosensors are presented in Table 1. Eight of the nine participants wore the E4 for at least four days outside of the clinic. One participant did not wear the E4 at all outside of the lab visits. The group wore the E4 for 270 days totaling 2, 405 full hours of physiological signals. The retention rate for wearing the biosensor in everyday life for up to 8 weeks (seven out of nine patients, 78%) was just under our criterion for success (80%), but 0.8 is within the 95% CI (0.4, 0.97) of our estimated retention rate.

**Table 1:** Descriptives for naturalistic data collection from adolescents (ages 10–16 years) with OCD.

**ID**	**Sex**	**Age group**	***N* Tags**	***N* hours**	***N* days**	**Mean hours/day**	**Mean *N* tags/hour**
0	M	10-12	26	233.32	36	6.48	0.11
1	M	10-12	313	372.09	35	10.63	0.84
3	F	10–12	9	29.03	4	7.26	0.31
4	M	10–12	6	336.71	35	9.62	0.02
5	M	13–16	38	118.65	29	4.09	0.32
6	F	13–16	73	419.34	47	8.92	0.17
7	F	13–16	238	521.33	48	10.86	0.46
8	F	13–16	936	374.93	36	10.41	2.50

During the observation period, participants registered 2,146 OCD events using the event tag button on the E4. Patients did not report any false or accidental registrations/ tags. Plots of number of tags per hour over the 8 week observation period show the frequency of OCD event tags of each participant (see Supplementary Figure S2). The frequency of tags appeared to decrease over the observation period for three participants. Two participants showed varied tagging behavior over the 8 weeks. OCD event tagging for two other participants was consistently low across the observation period with occasional increases in tagging.

The most represented OCD symptom types in our sample were forbidden thoughts, followed by symmetry, and then cleaning. Hoarding was rare, but a dominant symptom for one participant. OCD symptom types, counts, and severity for each patient assessed prior to data recording are displayed in Supplementary Table S1.

**Figure 4:**
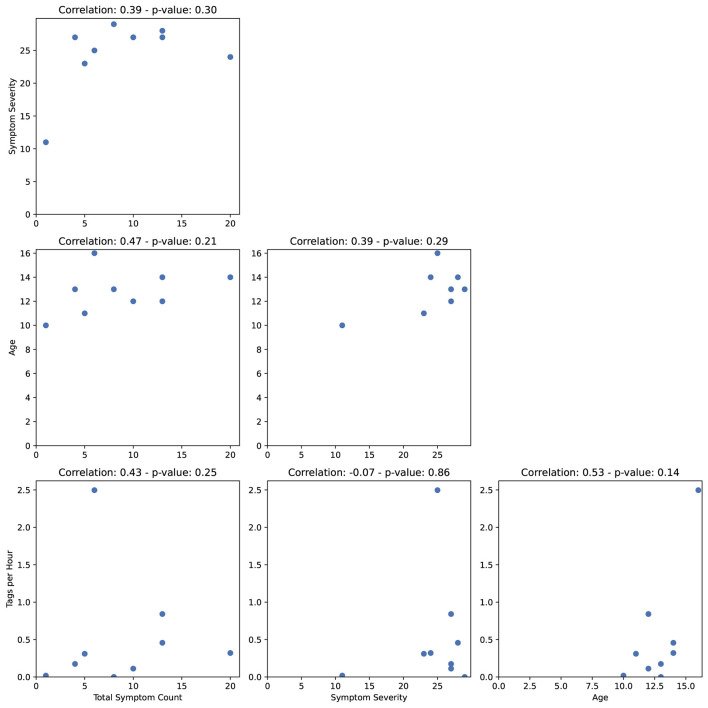
Spearman rank-correlations between the total symptom count, symptom severity, age, and number of tags per hour.

Figure 4 shows the correlations between the total symptom count, OCD symptom severity, age and number of tags per hour. None of the *p*-values were significant. However, there were medium-sized, positive correlations between the number of OCD event tags per hour and participant age as well as the total OCD symptom count. Additionally, there was a medium-sized, positive correlation between age and total OCD symptom count. The number of tags per hour was not related to OCD severity. However, positive, medium-sized correlations were found between number of tags per hour and the degree of interference from OCD symptoms (rho = 0.40, *p* = 0.25) and number of forbidden thought-related symptoms (rho = 0.71, *p* = 0.03). Negative, medium sized correlations were observed between distress due to OCD symptoms and number of physiological data hours per day (rho = −0.55, *p* = 0.12). All correlations between variables are displayed in Supplementary Table S2 and discussed in Section 4 of the Supplementary material.

### 3.2. Model performance

#### 3.2.1. Generalized predictive models

**Figure 5:**
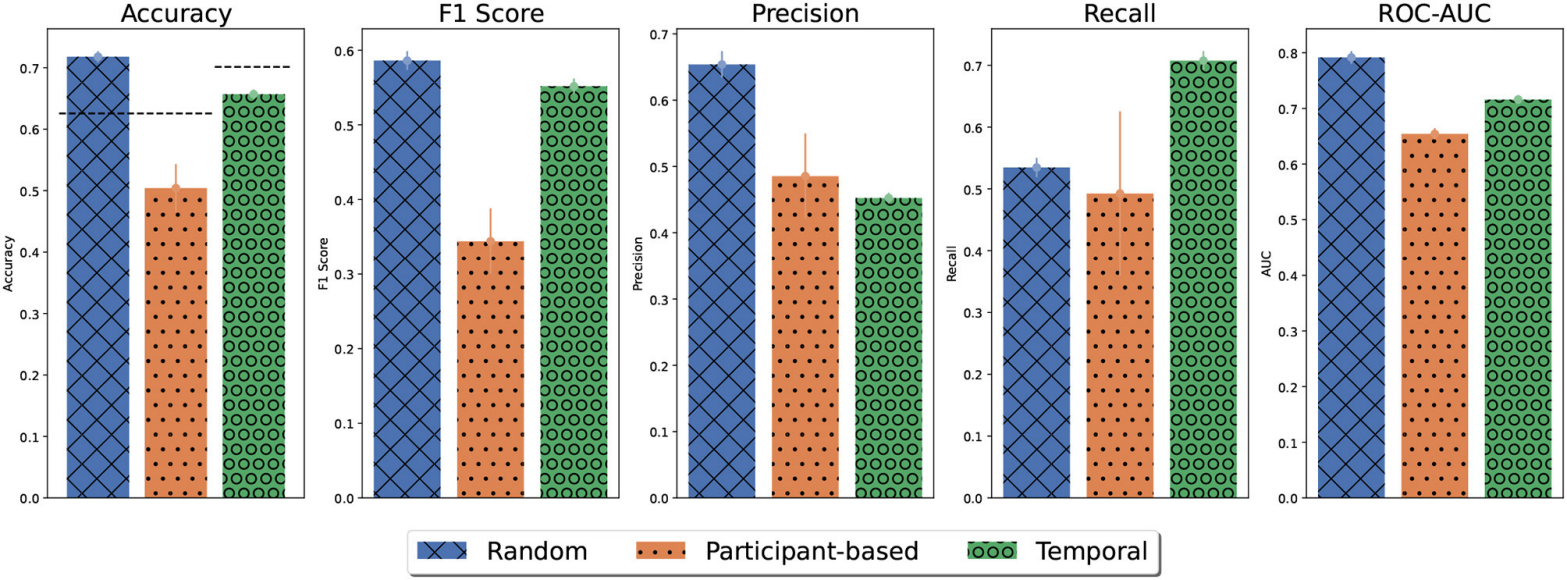
Average model performance for different cross-validation strategies. Error bars denote the standard error of the mean. For random and participant-based cross-validation, model performance was estimated using outer folds. For temporal cross-validation, model performance was based on 10 repetitions with a holdout set. For participant-based models, the performance results for each participant were weighted by their population percentage. The accuracy obtained by majority guessing is denoted by the dashed line.

In total, 1, 639 OCD events and 2, 739 nonevents were sampled from all participants. The performance of detecting OCD events using different cross-validation strategies (random split, participant split, and time-split) is shown in Figure 5. Using a 10-fold random cross-validation, the average test accuracy was higher than 70%. However, the F1-score was just under 60%, primarily due to low recall. The recall was 50%, indicating that only half of the tagged events were detected on average. The precision was 66% indicating that one in three positive predictions were false positives. The average AUC-ROC was 0.8, indicating that the classifiers generally ranked the tagged events higher than the negative observations. Thus, the detection threshold might be adjusted to increase the recall. Based on the ROC curves, the detection threshold can be adjusted to detect 90% of all tagged events at the cost of falsely detecting half of the negative observations (Supplementary Figure S3A). The performance measures of the participant-based cross-validation were all lower than the random cross-validation, indicating that the generalization across participants was poor. In contrast, the generalization to new data from the same participants of the temporal cross-validation was comparable to the performance of random cross-validation in terms of accuracy and F1 score. A notable difference was that the recall was higher and the precision was lower for the temporal cross-validation than for random cross-validation, indicating that more positive predictions were made using temporal cross-validation. This was also reflected in the temporal cross-validated ROC-curves (Supplementary Figure S3B). A standard detection threshold falsely predicted a third of the negative cases (non-OCD events) as positive cases (OCD events) while obtaining a recall of 70%. However, when the detection threshold was adjusted to obtain a recall of 90%, half of the negative observations were still falsely detected, similar to random cross-validation. Thus, while the generalization to newly recorded data was good, it made the models more prone to false positives.

**Figure 6:**
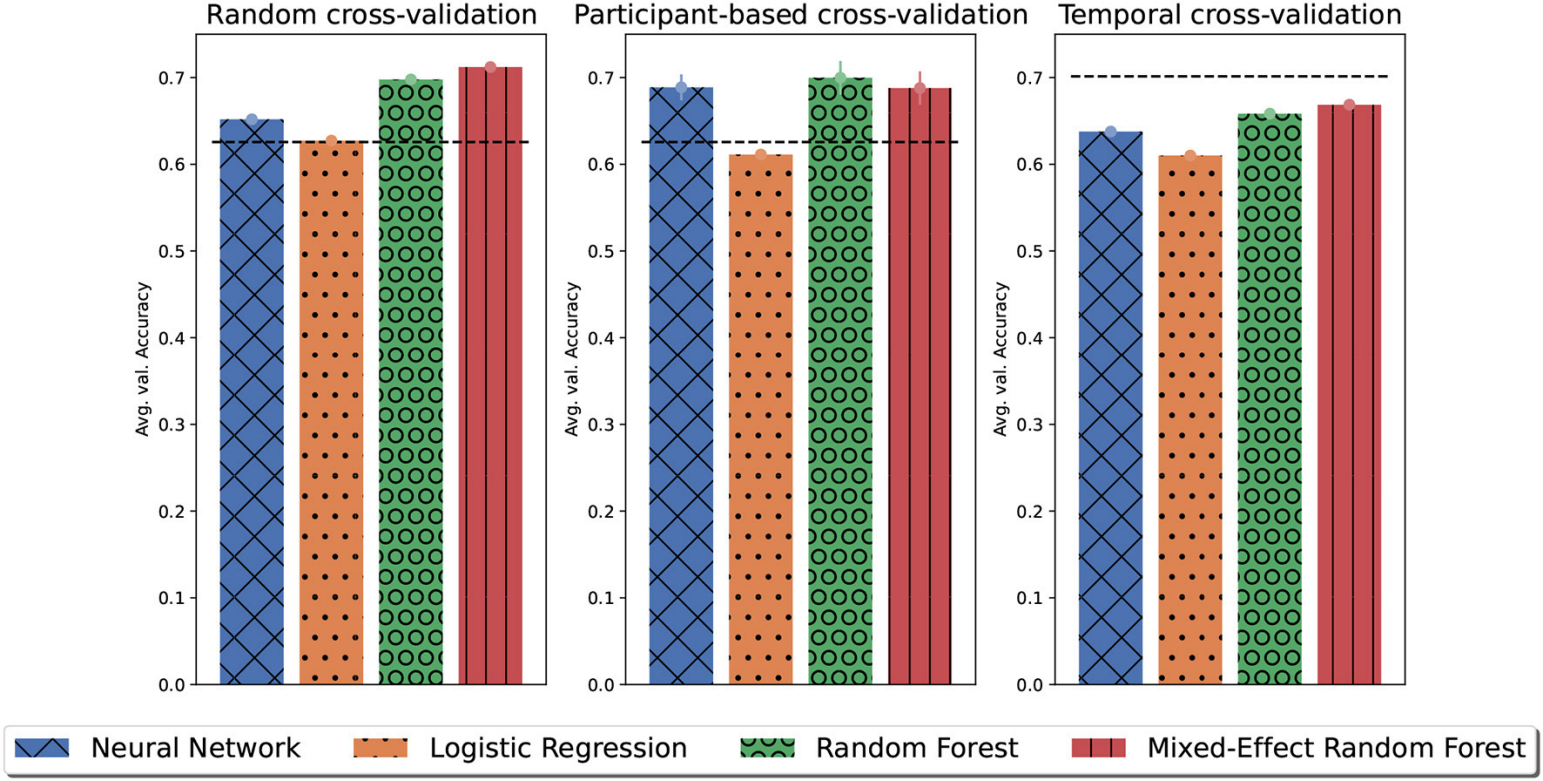
The maximum of the average validation accuracy for each model type on the inner-layer validation sets. Error bars denote the standard error of the mean. Dashed lines indicate the average value accuracy obtained by majority guessing.

Figure 6 shows the maximum average validation accuracy for each model type in the inner-layer validation sets. For all cross-validation strategies tree-based models achieved the highest accuracy. Particularly, the MERF model outperformed a normal random forest model for random and temporal cross-validation as the MERF model may have used between patient variations during predictions. However, for participant-based cross-validation, the MERF models had not previously seen data from the test participant and defaulted to a normal random forest classification without any hyperparameter optimization leading to relative worse performance.

#### 3.2.2. Personalized predictive models

The results per participant of personalized models averaged over 10 repetitions are displayed in Table 2. The accuracy of personalized models was below the accuracy obtained by simple majority guessing for all participants except one. The model accuracy for the remaining participant was similar to majority guessing. This indicates that even personalized models trained on a small training set with few tagged events predicted some OCD events. However, the F1-score for most of these personalized models, with <50 tagged events, was 0, meaning that all predictions were false positives. The only exception was Participant 0 who achieved an average recall of 0.8, which means that eight of the 10 models correctly identified the tagged event. Personalized models for participants with 313 and 238 tagged events correctly identified some tagged events. However, the models suffered from false positives and false negatives. For one of these participants, the models identified 20% of the tagged events on average and one in five of the predicted OCD events were correct. For the other participant, the results were slightly better. Sixty percent of all tagged events were detected on average, and two in five of the predicted OCD events were correct. For the participant with 936 tagged events, personalized models detected an average of 80% of all OCD events. However, the models still suffered from false positives, as every other OCD prediction was a false positive and the F1 scores indicated poor model performance overall due to the small number of observations available.

**Table 2:** Comparison of personalized and temporal generalized model performance per participant with oversampling of OCD events.

**Participant**	**0**	**1**	**3**	**4**	**5**	**6**	**7**	**8**
*N* train tags	25	296	9	4	37	72	223	867
*N* test tags	1	17	0	2	1	1	15	69
Accuracy								
Mean personal	0.84	0.57	0.68	0.87	0.80	0.83	0.54	0.56
Mean temporal	0.89	0.63	0.55	0.92	0.84	0.89	0.58	0.56
Mean temporal increase	0.06	0.06	−0.13	0.05	0.04	0.06	0.04	0.00
*p*-value	0.33	* < 0.01*	**0.01**	*0.01*	0.51	0.17	0.11	
F1-score								
Mean personal	0.51	0.47	0.00	0.00	0.00	0.00	0.23	0.61
Mean temporal	0.07	0.45	0.00	0.07	0.32	0.05	0.48	0.62
Mean temporal increase	−0.43	−0.02	0.00	0.07	0.32	0.05	0.25	0.01
*p*-value	**0.01**	0.35	–	0.19	*0.01*	0.17	* < 0.01*	0.41
Precision								
Mean personal	0.44	0.38	0.00	0.00	0.00	0.00	0.22	0.50
Mean temporal	0.04	0.42	0.00	0.05	0.24	0.03	0.39	0.50
Mean temporal increase	−0.39	0.03	0.00	0.05	0.24	0.03	0.17	−0.00
*p*-value	**0.02**	*0.03*	–	0.18	*0.03*	0.17	* < 0.01*	0.97
Recall								
Mean personal	0.80	0.59	0.00	0.00	0.00	0.00	0.24	0.77
Mean temporal	0.20	0.48	0.00	0.15	0.60	0.20	0.63	0.81
Mean temporal increase	−0.60	−0.11	0.00	0.15	0.60	0.20	0.39	0.04
*p*-value	**0.01**	**0.01**	–	0.19	*0.01*	0.17	* < 0.01*	0.21
Area under the ROC								
Mean personal	0.88	0.62	0.00	0.12	0.61	0.46	0.46	0.58
Mean temporal	0.92	0.68	0.00	0.79	0.85	0.64	0.57	0.57
Mean temporal increase	0.04	0.06	0.00	0.67	0.24	0.18	0.11	−0.01
*p*-value	0.58	* < 0.01*	–	* < 0.01*	* < 0.01*	*0.05*	* < 0.01*	0.11

Note. Significant *p*-values of a two-tailed t-test for a difference in means are denoted by italics when temporal generalized models have better performance or bold when personalized models have better performance.

#### 3.2.3. Comparative model performance

**Figure 7:**
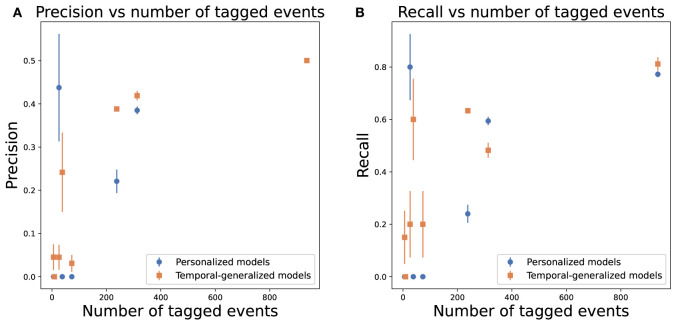
Average precision and recall over 10 repetitions, as a function of the number of tagged OCD events, per adolescent of personal (blue) and temporal generalized (orange) models. Error bars denote the standard error of the mean. **(A)** Precision. **(B)** Recall.

The personalized predictive models were compared to temporal generalized models. The per participant results of temporal generalized models and personalized models averaged over 10 repetitions are displayed in Table 2. For all participants, except one, temporal generalized models gained a small increase in accuracy compared to personalized models. However, this increase was only significant for two participants (1 and 4). For another participant (3), the accuracy of the temporal generalized model decreased significantly compared to personalized models. These results suggest that the temporal generalized models classified more events as OCD events that were actually non-OCD events, i.e., false positives. For most participants, temporal generalized models made more positive predictions than personalized models. Temporal generalized models marginally improved performance in terms of precision and recall for most patients with few tagged events. Figure 7 summarizes the relationship between the number of tagged events and precision and recall for temporal generalized models and personalized models, Precision and recall increased significantly for Participants 5 and 7 and slightly for Participants 4 and 6. However, given the small amount of positive observations or OCD events in the test set for these participants conclusions cannot be drawn. Moreover, for Participant 1, recall decreased and precision increased significantly as the temporal generalized models made fewer positive predictions. Similarly, precision and recall for patient 0 decreased significantly due to fewer positive predictions.

AUC-ROC values showed a significant increase when temporal generalized models were used for all patients except for Particitant 0, in which the increase was minor and for Participant 8, in which there was a non-significant decrease.

**Figure 8:**
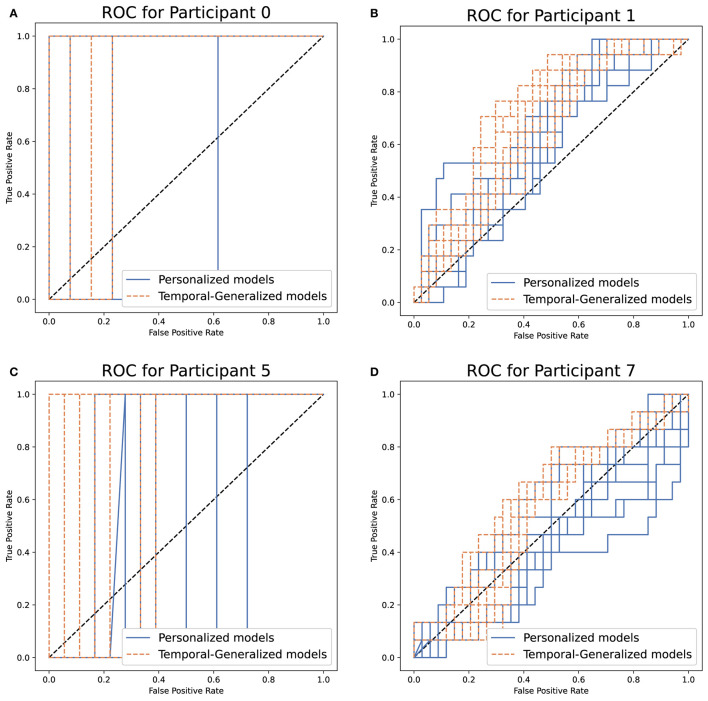
Ten repetitions of the receiver operating characteristics (ROC) for detecting OCD events in adolescents using personal (blue) and temporal generalized (orange) models. **(A–D)** Participant 0, 1, 5, and 7.

Figure 8 illustrates the receiver operating characteristics for patients 0, 1, 5, and 7. The average false positive rate required to detect most OCD events was reduced when using temporal generalized models compared to personalized models. Particularly, for Participant 0 (Figure 8A), temporal generalized models correctly classified the tagged OCD event in the test set with an average false positive rate of 7.7%. However, personalized models had an average false positive rate of 11.5%. For Participant(s) 0 (Figure 8A), the tagged OCD event in the test set would be among the first three predicted events for all temporal generalized models. Although improvements were generally seen for all patients, the performance for Participant 8 was virtually unchanged. We hypothesize that this was because the temporal generalized models were being dominated by this participant due to their relatively larger amount of data.

**Figure 9:**
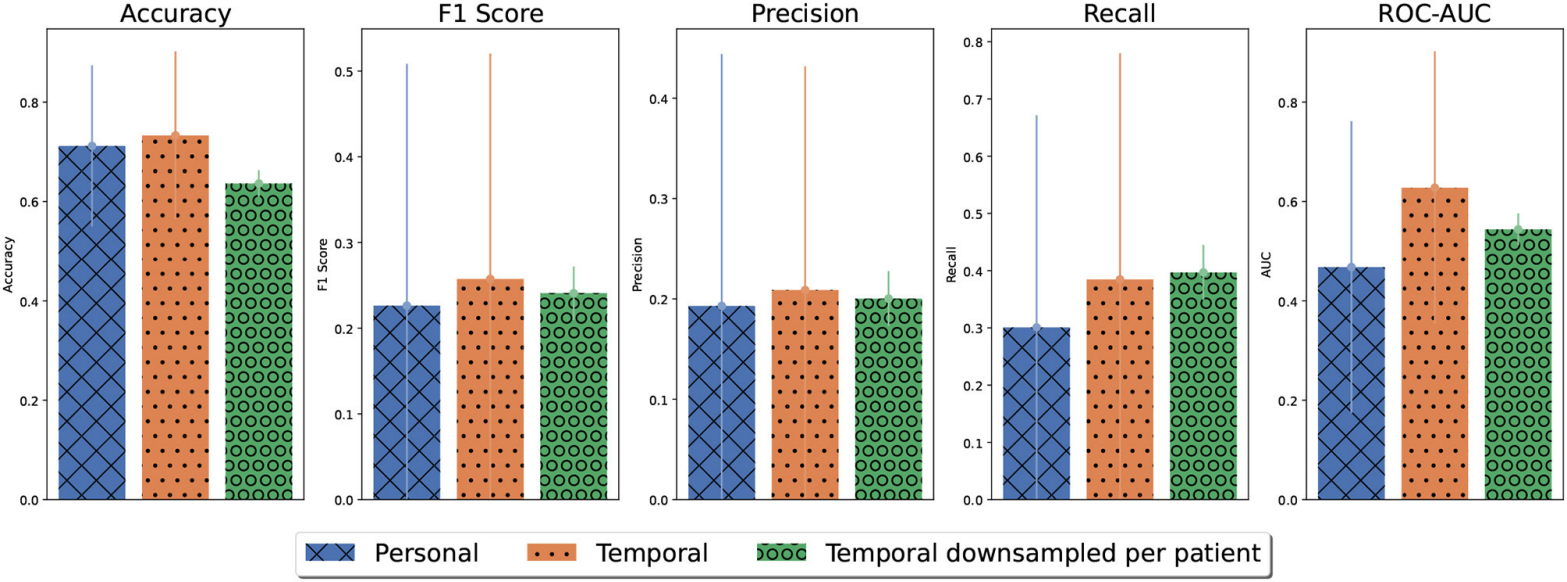
The average performance for detecting OCD events per participant of personalized, temporal generalized, and temporal generalized models, in which the training data from other participants has been down-sampled to match the data amount from the participant under evaluation.

Figure 9 shows the average performance per patient of personalized and temporal generalized models, with and without down-sampling of data from other patients to match the amount of data from the participant under evaluation. Down-sampling resulted in a boost in F1-score as seen when we included all data from other participants. However, down-sampling did not achieve a similar performance in accuracy or AUC-ROC. Moreover, the variation in performance between repetitions was greatly reduced using down-sampling of each participant, suggesting that performance was more consistent across patients albeit slightly worse on average.

### 3.3. Feature importance

To gain a better understanding of how the predictive models arrived at their classification decisions for OCD/ non-OCD events, we obtained SHAP values for the random cross-validation model. The SHAP values are derived from the models selected in the inner layer. For the random models, MERF was selected in all 10 folds of the inner layer and, thus, the SHAP values were derived from MERF classification. **Figure 11** shows the SHAP values of the 20 most important features. Features related to the slope of the BVP signal were the most important features, followed by features related to the frequency content of the BVP signal and the variation of the BVP signal. Although other cross-validation strategies can affect the order of these the most important features were consistent across the three validation strategies: features related to the slope of the BVP, the frequency content of the BVP, and the variation of the BVP. The SHAP values for participant-based and temporal generalized models are presented in Supplementary Figures S4, S5. The plots also show that high (orange dots) and low (blue dots) values of features had the same probability for predicting an OCD event. This illustrates the difficulty of predicting OCD events in-the-wild and highlights the need for nonlinear multivariate models.

### 3.4. Power analysis

**Figure 10:**
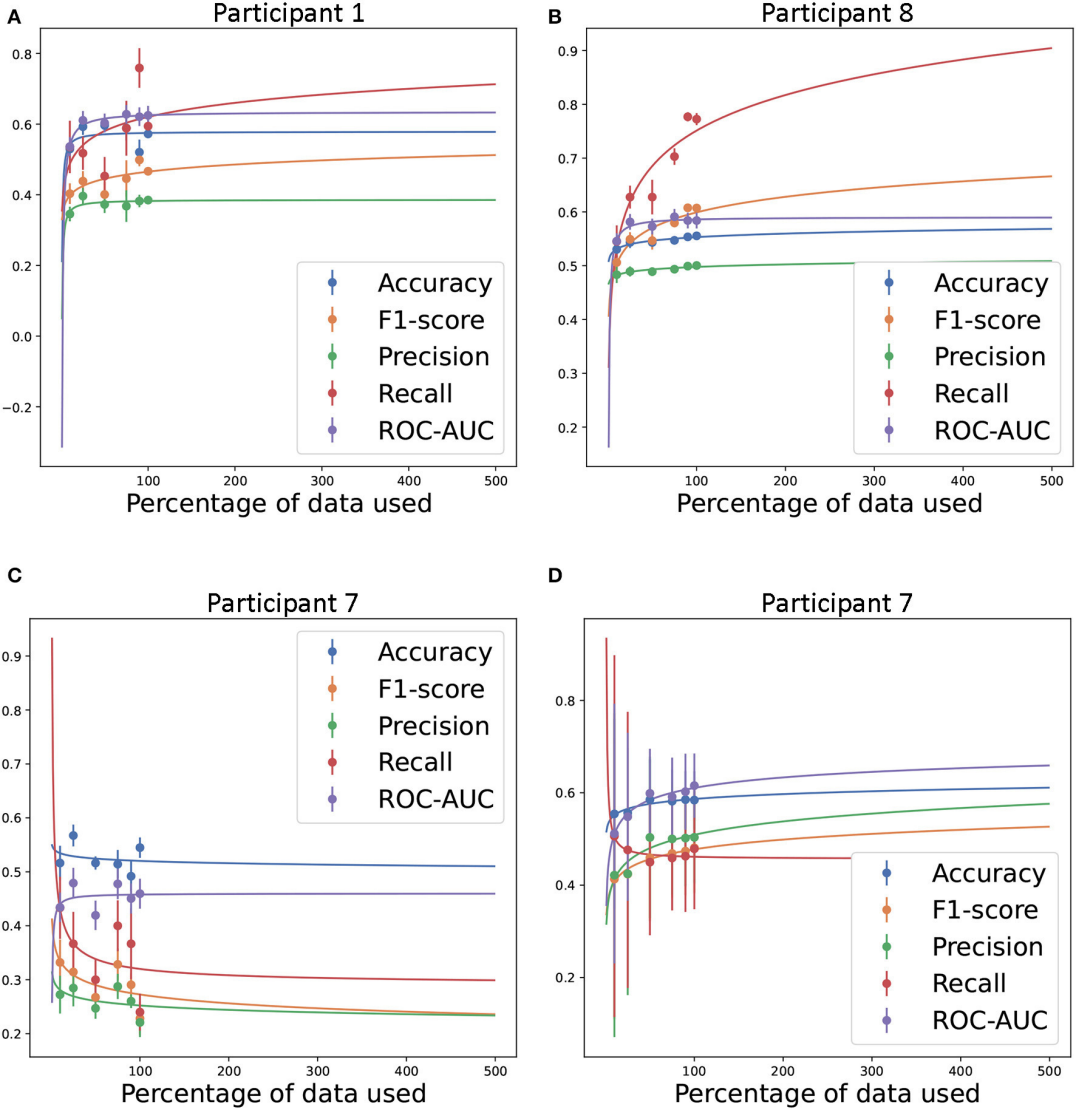
Estimated amount of training data required to achieve the given performance level in accuracy, F1-score, precision, recall, and ROC-AUC. Inverse power law fit to the performance levels obtained by using personal predictive models for Participants 1 **(A)**, 7 **(C)**, and 8 **(B)** (34) and random cross-validation for Participant 7 **(D)**. In all personal predictive models, the test set was held constant regardless of the amount of training data. In random cross-validation the testset was selected randomly from the sampled data. 100% training data equals 523, 754, and 1, 954 for Participants 1, 7, and 8, respectively. Experiments were repeated 10 times.

We down-sampled the amount of training data on personalized models for the three participants with the most observations to ensure sufficient OCD events in the test set (see Table 2). Figure 10 shows the evolution of the performance metrics for these three participants as a function of the percentage of training data used for training. For Participant 1 and 8 (Figures 10), the accuracy, ROC-AUC, and precision quickly reached their asymptotic values suggesting that increasing the amount training data would not improve these performance metrics. However, the recall and F1-score increased with more training data. Together, these performance metrics suggest that these models could learn to detect more OCD events with more training data, but the test set had some nonevents that become false positives with increased sensitivity. For Participant 7, performance metrics reached asymptotic values using 10% of the training data. Indeed, the curve fit for accuracy, F1-score, precision, and recall decreased with additional training data. However, this decrease may be a random effect as the asymptotic values had been reached. As discussed in Section 3.2.3, model performance increased for Participant 7 when training data from other participants was included using temporal generalized models. This indicates that for some individuals additional data was not sufficient to improve performances and data from other participants may be needed. One reason for this could be a significant change in the data between training and test sets perhaps due to illness or a change in the environment of the participant at test. Using random cross-validation for Participant 7, the performance metrics generally improved with additional data as expected. The maximum recall was still obtained using only 10% of the data. However, the variance from repeated experiments was high indicating that there was significant change in the data for this participant. One explanation for the change in data may be that frequency of registering OCD events changed over the observation period as shown in Supplementary Figure 1.

**Figure 11:**
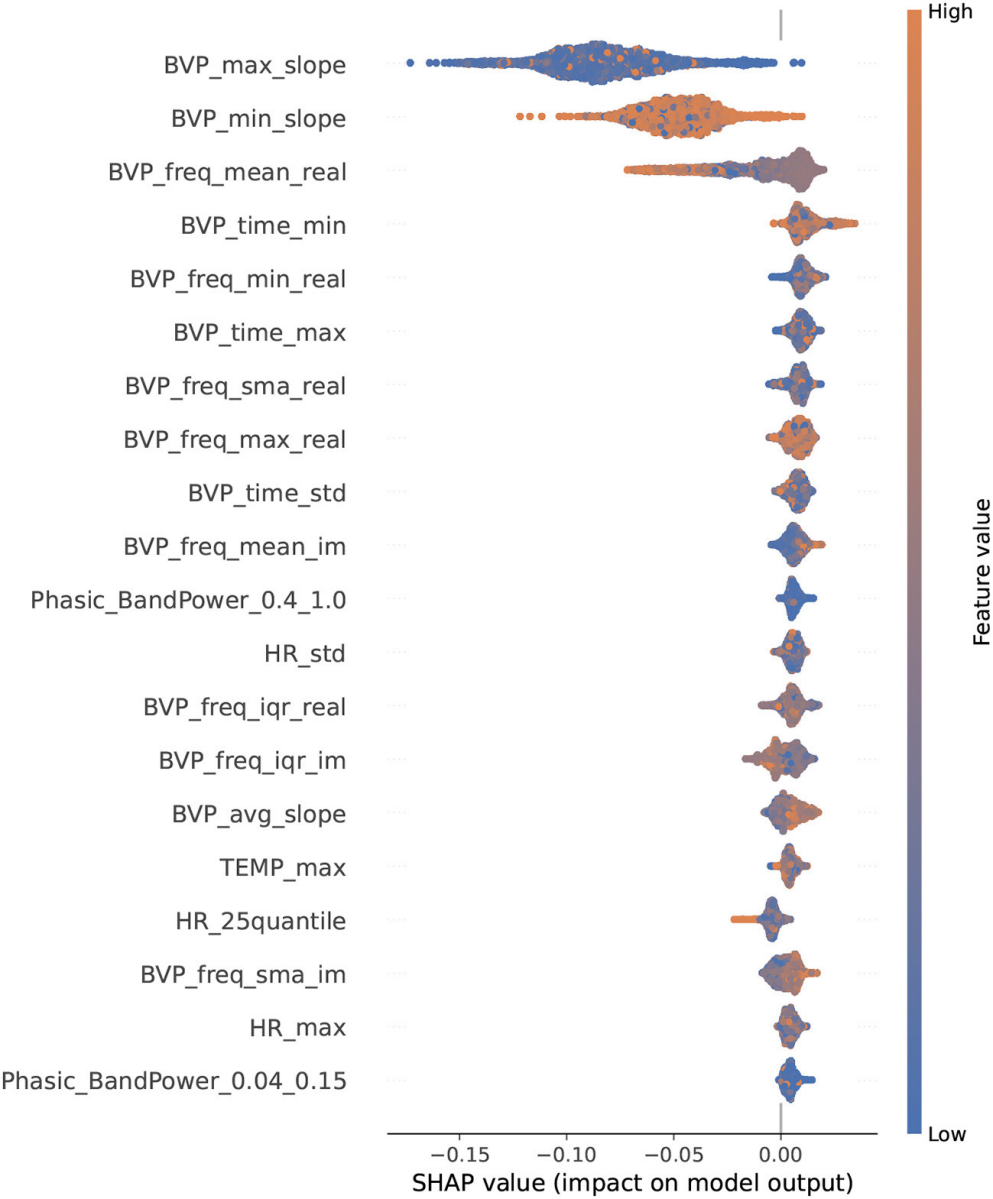
SHAP-values for the 20 most important features in the random cross-validation. SHAP values were derived from MERF models selected in the inner layer. Features are listed along the *Y*-axis in order of magnitude of impact on model predictions.

### 3.5. Comparison of features: rest vs. exposure

**Figure 12:**
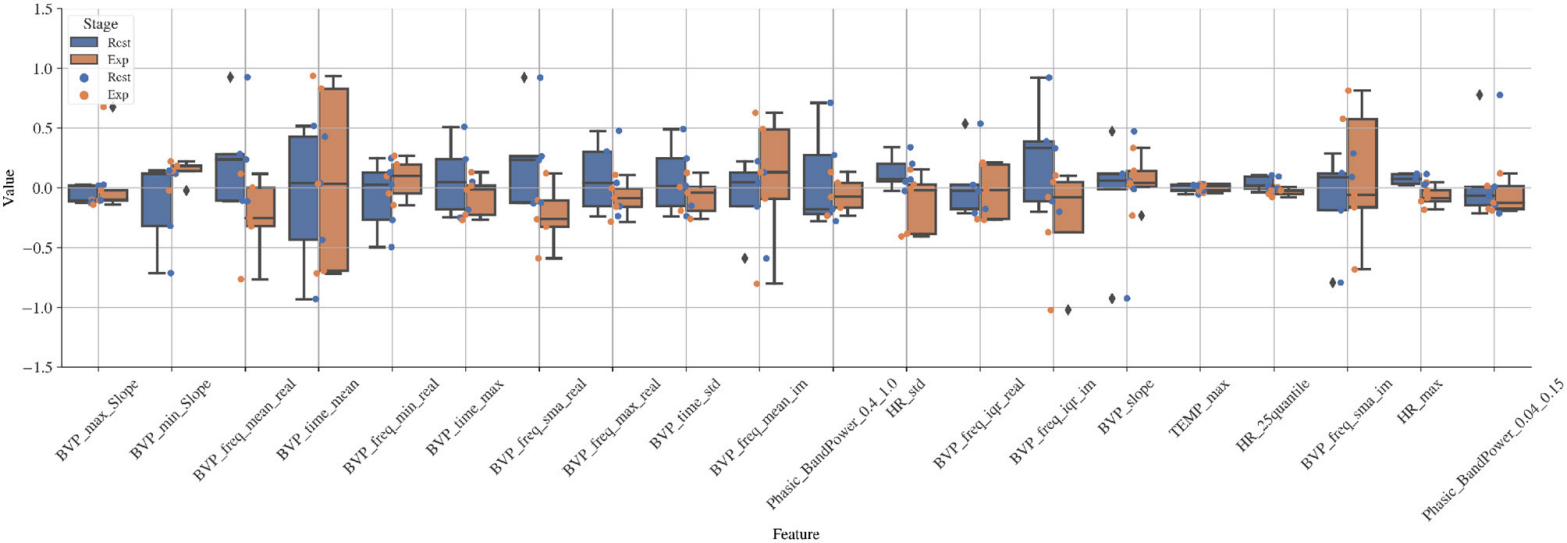
Boxplot of values of important features obtained from the SHAP analysis in random cross-validation (Figure 11) for rest and exposure conditions in the lab.

The box plot in Figure 12 displays group differences in the most influential features in the prediction models under conditions of rest and exposure in the lab. For a few of the features (related to BVP slope and frequency content), the box plots show differences between conditions. Generally, the raw signals did not differ visually between conditions (see Supplementary Figure S6). Heart rate in the rest condition was higher than in the exposure condition for the group, but HR features were not among the most influential in the predictive models from the in-the-wild data. This was supported by additional plots (shown in Supplemental Figure S8). Note that on the individual level, changes in heart rate differed across individuals (see Supplemental Figure 9). For some individuals heart rate was higher in the exposure condition compared to the rest condition toward the end of the five minutes. For one individual, heart rate was markedly higher in the exposure condition compared to the rest condition after one minute, but higher in the resting condition after three minutes.

## 4. Discussion

This feasibility study explored whether OCD episodes can be detected in the everyday lives of adolescents with OCD using physiological signals from a wrist worn biosensor. Previous studies have used wearable biosensors to capture OCD-related behavior in highly controlled settings or using several conspicuous sensors (13, 17). OCD episodes were defined as any time the individual felt stressed by their OCD symptoms. To be able to detect OCD episodes, children and adolescents with OCD would need to be willing to wear the biosensor during their everyday lives. The retention rate for wearing the biosensor in everyday life for up to 8 weeks was just under our criterion for success. Of the nine included participants, one refused to wear the wristband outside of the clinic and one dropped out of the study after four days of wearing the biosensor. In one case, symptoms of Asperger syndrome may have played a role in the resistance to wearing the biosensor. Given that we only have one case example and another study managed to collect an average of 10 consecutive days of E4 data from 20 adolescents (6–17 years) with autism in inpatient care, exploring the use of wrist worn biosensors in adolescents with autism spectrum disorders is still relevant. In the other case, the adolescent was concerned about the biosensor revealing information about them that they did not want to reveal. Such privacy concerns can be addressed in future trials by explaining more clearly how the biosensor and machine learning methods work.

The eight participants who wore the biosensor, wore it for an average of 33.75 days and 8.5 h per day. This rate of 60% of the total number of days of observation exceeded our success criterion of at least 30%. This rate reflects the participants' willingness to wear the biosensor, and thus, the tolerability of the biosensor, as well as missed days due to logistical issues with exchanging biosensors full of data for empty ones. Nonetheless, the adolescents in our sample with OCD, wore the biosensor longer than adolescents of a similar age in a weight management study (35). These results provide preliminary support that adolescents with OCD are willing to wear a biosensor to monitor OCD symptoms for an extended period of time. To use the biosensor as an objective measure of OCD-related distress or time occupied by OCD symptoms, which comprise four items on the gold standard measure of OCD symptom severity (CY-BOCS), the adolescents in this study wore the biosensor for more than enough time. The CY-BOCS requires respondents to estimate how much obsessions/compulsions caused distress and occupied their time in the past week on a likert scale (6). Most adolescents in our study demonstrated that they could wear the biosensor for seven consecutive days. A number of hypotheses can be drawn from our exploratory study. We found a medium sized, yet non-significant, negative correlation between distress due to OCD symptoms and number of biosensor signal hours per day. Future studies with sufficiently large samples may test the hypothesis that youth who report higher levels of distress in the OCD symptom severity interview (CY-BOCS) wear the biosensor less than adolescents with lower levels of OCD distress.

Participants tagged an average of 205 OCD events over the entire period with nearly 6 tags per day. The frequency of tags appeared to decrease over the observation period for three participants. The frequency with which participants tagged OCD events varied within our sample. OCD event tagging for two participants was consistently low across the observation period with occasional increases in tagged events. Two other participants showed varied frequency of event tagging over the 8 weeks. None of the participants reported accidentally pressing the event mark button to tag an OCD event (i.e., a false positive). In our sample, adolescents who were older, endorsed more forbidden thought-related symptoms, endorsed more OCD symptoms in general and rated symptoms as more functionally impairing tended to tag more OCD events per day. The association between time occupied by OCD symptoms and number of OCD events per day was smaller. These preliminary findings support the clinical phenomenology of OCD in youth that number of symptoms are not associated with the time occupied by OCD symptoms as reported in clinical interviews (36). The associations between age, symptom load, time spent on OCD and frequency of OCD symptom disturbance registered in real time is worth testing in a larger sample. Hence, more objective measures of symptom load and time occupied with OCD may be plausibly developed in the near future.

Our preliminary results suggest that detection of OCD events in the wild is possible. We compared four models' ability to classify OCD-episodes: a neural network, logistic regression, random forest and mixed-effect random forest. The tree-based models (RF and MERF) demonstrated the best performance and reached our success criterion for feasibility of 70% accuracy. Models performed better when generalizing across time than generalizing across patients. However, generalized temporal models trained on multiple patients outperformed personalized models trained on a single patient. Furthermore, generalization to new times made models more prone to false positives. Given the current levels of false positives, these models cannot be applied to the task of delivering momentary interventions, but these models may be useful in assessing treatment progress.

Future attempts will benefit from finding strategies for reducing the number of false positives. Apart from training the models on data from more patients, one possible way of reducing the number of false positives is including training data from control participants without an OCD diagnosis. Our preliminary results also suggest that future studies aiming to detect OCD events in newly recorded data will require a large number of labeled OCD-episodes or a different modeling approach. For example, a time-series approach would retain more data and may better account for the temporal variation. A study predicted aggression in 20 adolescents with autism using a time-series approach with promising results (16). Notably, the study also used behavioral observations to mark the start and stop times of aggressive events (16). Measuring the duration of events has the advantage of more precision in when the event of interest is occurring and when not. However, it is also more labor intensive than pushing a button once.

The most influential features in our models included features related to the slope, the frequency content of the BVP, and the variation of the BVP. Our comparison of these features under controlled conditions of rest and exposure showed differences in features related to slope of the BVP, the frequency content of the BVP, and the variation of the BVP, supported the feature importance results. In some instances the direction of the effects were in opposite directions, which could be expected when comparing non-linear modeling with univariate box plots. Surprisingly, the raw heart rate signal in a subsample (*n* = 5) of our participants tended to be higher during conditions of rest than during exposure (Supplementary Figure S8). We would expect the exposure to be more stressful and result in a higher heart rate. However, the sample size was small. For some individuals at certain times during the five minutes, heart rate was higher during exposure than during rest. Still, we cannot rule out that events preceding the resting period did not contaminate the resting period or that. Some of our participants found the resting period, in which they were asked to sit quietly and still, more stressful or arousing than the exposure task. Alternatively, this unexpected result may be due to only using the first 5 min of exposure. This preliminary result is difficult to compare with previous findings due to the scarcity of research in this area and methodological differences. A previous pilot study observed higher heart rate during exposure than during benign activities and recovery in five adolescents with OCD (17).

Although it was not possible to perform a traditional sample size calculation to plan future studies that aim to detect OCD or similar events, we performed a power analysis that investigated the amount of training data (events and nonevents) that would be needed to adequately train classification models to differentiate OCD events from nonevents. The results of these analyses were inconclusive, thus, we are unable to recommend a specific training set size for future studies. However, these analyses indicate that for some individuals more training data would improve model performance. For others, the low quality of the data hampered accurate classification. Low data quality may have stemmed from changes in participant behavior, as observed by changes in frequency of registered OCD events, and changes the environment that impacted the physiological signals such as illness, vacation, and seasonal changes. In these cases, transfer learning may improve classification, in which data from other individuals is applied to individual problematic data. Transfer learning will require a large sample of participants and observations. In general, more information is needed about nonevents, such as periods of physical exertion or non-OCD related stress.

This study had limitations that should be taken into account. The size of our sample does not allow any conclusions to be drawn from statistical analyses or machine learning model performance, other than toward its feasibility. Another limitation of the design of the study was that we did not collect systematic information about skin tone. A previous study has demonstrated that the PPG sensors with green light, as the one used in the present study, had increased measurement error with darker skin tones (37). Thus, any future study should systematically collect information of skin tone and if possible adjust for or at least assess the measurement error this introduces. A third limitation was the uncertainty of the duration of OCD events. Participants marked events as a single moment in time by pressing a button and we set a 5 min window before the marked time points. This labeling technique was not labor intensive for the participants or the researchers. Future studies may weigh the advantages of obtaining more precise measures of OCD event duration by asking participants to register more detailed information about OCD events.

## 5. Conclusion

Preliminary results from machine learning modeling using physiological signals as input suggest that OCD events can be distinguished from non-OCD events in the the daily lives of adolescents. The results of this feasibility study have produced hypotheses to be addressed in larger studies. One set of hypotheses could involve specific OCD symptoms, in terms of content and form, that can be measured with a wearable biosensor. Future studies would also benefit from investigating whether specific clinical presentations are contraindicated for using wrist worn biosensors for symptom monitoring. Future modeling endeavors may benefit from more registered OCD events and a time-series modeling approach, which will require more certainty in data labels.

## Data availability statement

The raw data supporting the conclusions of this article will be made available by the authors, without undue reservation.

## Ethics statement

The studies involving humans were approved by Ethics Committee of the Capital Region of Denmark on June 17, 2021 (ref. nr. H-18010607-79689). The studies were conducted in accordance with the local legislation and institutional requirements. Written informed consent for participation in this study was provided by the participants' legal guardians/next of kin.

## Author contributions

NNL, AKP, and LKHC designed the naturalistic and lab studies. NNL and ARCMJ carried out the studies and collected the data. AKP oversaw any issues with adverse events. KVO, SD, NNL, and LKHC planned the data analysis. KVO analyzed the in-the-wild-data. SD analyzed the lab data. LKHC and NNL supervised the analyses. NNL and KVO wrote the first draft. All authors have edited and approved the manuscript.
