# Supplementary material for: Predicting obsessive-compulsive disorder episodes in adolescents using a wearable biosensor—A wrist angel feasibility study
Source: Front Psychiatry. 2023 Oct 2;14:1231024. doi: 10.3389/fpsyt.2023.1231024 (PMC10578443; doi:10.3389/fpsyt.2023.1231024)
Supplement: Supplementary file 1 [file Data_Sheet_1.pdf]

# Supplementary Material

## 1 TABLE OF CONTENTS


- Section 2: Tagged OCD events per hour over days and weeks
- Section 3: Sample OCD symptom characteristics
- Section 4: Correlations between participant characteristics and use of biosensor
- Section 5: Filtering of physiological data
- Section 6: Signal preprocessing and feature extraction
- Section 7: Performance metrics for evaluation
- Section 8: Selected models and hyperparameters
- Section 9: ROC-AUC for random-split and time-split cross-validation
- Section 10: SHAP-values for the most important features
- Section 11: Comparison of features under controlled conditions
- Section 12: Power analysis
- Section 13: Tables
- Section 14: Figures


## 2 TAGGED OCD EVENTS PER HOUR OVER DAYS AND WEEKS

To explore participant use of the biosensor by hour, hours per day and per week, we calculated the mean number of hours of physiological data recorded per day and the mean number of tagged events per hour for each participant. The left side of Figure S1 provides an overview of how often participants tagged OCD events (blue dots) over days of the observation period in relation to meetings with researchers (denoted by red lines). Large gaps in tagged events (blue dots) reflect periods when the participant did not have a biosensor (e.g., see Participant 8). The right side of Figure S1 shows how tagging OCD events varied across participants and within participants over time.

## 3 SAMPLE OCD SYMPTOM CHARACTERISTICS

To understand the content, form and number of the participants' obsessions and compulsions and how they may relate to wearing a biosensor and tagging OCD events, we examined the checklist of the CY-BOCS (Goodman et al., 1991) and grouped symptoms based on previous work (Leckman et al., 1997; Bloch et al., 2008). The top half of Table S1 summarizes the number of OCD symptoms in each category and in total endorsed by each participant before the observation period (T1). The bottom of Table S1 summarizes OCD severity as well as pairs of obsession and compulsion severity items for time occupied by symptoms (items 1a and 6a), interference caused by symptoms (items 2 and 7), distress caused by symptoms (items 3 and 8), resistance against symptoms (items 4 and 9), and control over symptoms (items 5 and 10) (Goodman et al., 1991).

# 4 CORRELATIONS BETWEEN PARTICIPANT CHARACTERISTICS AND USE OF BIOSENSOR

To explore the relationships among participant characteristics and biosensor data, we calculated Spearman rank-correlations between number and type of OCD symptoms, symptom severity (subscales and total), age, amount of recorded data, and number of tagged events. This was an exploratory analysis to generate hypotheses for studies with more power. Table S2 summarizes the correlations.

# 5 FILTERING OF PHYSIOLOGICAL DATA

Although participants were asked to wear the biosensor during waking hours, the data indicated that some participants wore the biosensor during sleep or that the biosensor was left turned on after removal at night. Thus, the biosensor signals were filtered to remove periods when the E4 was on while the patient was sleeping or the patient was not wearing the E4. Figure S2 shows a recording of more than $50$ hours from a wristband containing periods when the participant was likely sleeping or not wearing the wristband. During the first day the wristband was removed shortly before $11:00$ but not turned off. The subsequent recording was visibly different from periods when the wristband was worn. The heart rate averaged around $140$, the EDA was steady at $0$, the temperature dropped to what was plausibly room temperature, approximately $20$ degrees Celcius, the BVP was steady at $0$, and the acceleration was constant in all three dimensions. Periods when the wristband was not worn were removed by placing a threshold on the temperature and EDA signals since these had the same sampling rate and differed the most from ordinary recordings. Measurements with a temperature less than $25$ degrees Celcius and an EDA less than $0.01$ were removed. This filter also removed short periods during the second day when the wristband was removed for one and two hours.

Probable periods of sleep were removed manually. To identify recordings possibly containing periods of sleep, we looked for recordings, which ended on a later date than the start date or recording with a duration longer than $16$ hours. Using this method, we identified $28$ potential cases of sleep for manual filtering. Manual filtering of sleep periods were done based on the BVP and acceleration signals, which were visibly different during sleep. A second night is displayed in Figure S2. As shown in Figure S2, sleep periods often contained tagged events. We did not wish to remove these tagged events unless we were $100\%$ certain that they were false tags. Thus, when possible, we filtered sleep periods starting after the last tag and ending $5-10$ minutes before the first tag in the morning. There were two cases in which we diverted from this principle.

After filtering periods of sleep and time, in which the wristband was not worn, we split the recorded data into segments. The data was split whenever the time difference between two consecutive timestamps exceeded five minutes. The resulting segments were considered independent recordings for downstream feature extraction.

- In one recording, the first tag of the morning was a series of six tagged events within $15$ seconds. We deemed this to be an attempt to turn the wristband on. Therefore, we ignored these six tags and ended the sleep filtering just after the tags.
- In another recording, the wristband was turned on late at night and a tagged event occurred only $30$ seconds into the recording. We deemed this window too short to extract meaningful features and removed the tag.

## 6 SIGNAL PREPROCESSING AND FEATURE EXTRACTION

Descriptions of the extracted features are displayed in Table S3. All extracted features are standardized to zero mean and unit variance within each training fold of the cross-validation. The mathematical definitions of these metrics are available in our analysis plan Olesen et al. (2023).

#### *6.0.0.1 Blood volume pulse (BVP)*

Before features could be extracted from the five-minute windows of the BVP signal, we had to remove noise. Noise reduction of the BVP signal was done within five-second segments (Fromberg, Das, and Clemmensen, 2023). Thus, within each sampled five-minute window, the recorded BVP was further segmented using a rolling window of five seconds with a time step of one second (Fromberg et al., 2023). We determined the noise level based on skewness and kurtosis for each segment(Krishnan, Natarajan, and Warren, 2010). If the kurtosis was less than $\epsilon_k = -0.5$ and the magnitude of the skewness was less than $\epsilon_s = 1$, the segment was deemed a low noise segment and useful for feature extraction. In each low-noise segment, we detected systolic peaks using the NeuroKit2 library (Makowski, Pham, Lau, Brammer, Lespinasse, Pham et al., 2021). These peaks were used to calculate the interbeat interval and the successive differences of this interval. Then, we computed the average interbeat interval (R-R interval) and root mean square of successive differences (RMSSD) using all low-noise segments within the five-minute window. Additionally, in each low-noise segment, we computed the time-domain features; mean, standard deviation (SD), median, minimum, maximum, and slope as well as the frequency-domain feature; mean, SD, median, interquartile range, minimum, maximum, and sum of frequencies. The frequency-domain features were split into real and imaginary components. All features were averaged across the low noise segments within each five-minute window for the final set of features. Finally, we included the minimum and maximum slopes for a low-noise segment as features.

#### *6.0.0.2 Heart rate*

The heart rate was calculated from the BVP signal using a proprietary algorithm from Empatica. From the heart rate, we computed the following features within each sampled five minute window: mean, SD, minimum, $25\%$ quantile, median, $75\%$ quantile, maximum, interquartile range, and slope.

#### *6.0.0.3 Skin temperature*

The skin temperature was preprocessed using a sixth-order Butterworth low-pass filter with a cut-off frequency of 1Hz. In each sampled five minute window, we extracted the following features from the skin temperature: mean, standard deviation, minimum, maximum, and slope.

#### *6.0.0.4 Electrodermal Activity*

The EDA signal was first pre-processed using a sixth-order Butterworth lowpass filter with a cut-off frequency of 1 Hz and normalized to the interval $[0, 1]$. The normalized signal was decomposed into its tonic and phasic parts using the NeuroKit2 library (Makowski et al., 2021) for Python. In addition to the tonic and phasic components, the unnormalized EDA signal was retained for feature extraction as normalization may inadvertently remove explanatory features related to the level of the signal. From the tonic component, we calculated the minimum, $25\%$ quantile, median, $75\%$ quantile, maximum, interquartile range, and slope within each five-minute window. Features extracted from the phasic components included mean, standard deviation, number of peaks, average peak amplitude, average response time, and power in the frequency bands ultralow frequency (ULF: 0.01-0.04 Hz), low frequency (LF: 0.04-0.15 Hz), high frequency (HF: 0.15-0.4 Hz), and ultra-high frequency (UHF: 0.4-1.0 Hz). From the unnormalized signal, we computed

mean, standard deviation, minimum, $25\%$ quantile, median, $75\%$ quantile, maximum, interquartile range, and power in the frequency bands above.

## 7 PERFORMANCE METRICS FOR EVALUATION

We used the following four performance metrics to evaluate models

$$\text{Accuracy} = \frac{TP + TN}{TP + TN + FP + FN} \tag{S1}$$

$$\text{Precision} = \frac{TP}{TP + FP} \tag{S2}$$

$$\text{Recall} = \frac{TP}{TP + FN} \tag{S3}$$

$$\text{F1-Score} = \frac{2 \cdot \text{Precision} \cdot \text{Recall}}{\text{Precision} + \text{Recall}} \tag{S4}$$

$TP$ (true positive) represents the number of correctly classified OCD events, $TN$ (true negative) represents the number of correctly classified non-OCD events, $FP$ (false positive) represents the number of falsely classified OCD events, and $FN$ (false negative) represents the number of falsely classified non-OCD events, respectively.

Additionally, we calculated the receiver operating characteristic (ROC) curve to plot the recall against the false positive rate (FPR) $\frac{FP}{FP+TN}$ at various threshold levels. The ROC curve is a graphical plot that illustrates the performance of a classifier as its discrimination threshold is varied. We used the area under the curve (AUC) metric to evaluate the performance of the models. The AUC is a metric that measures the overall performance of the classifier, taking into account different discrimination thresholds. The AUC score ranges from 0 to 1, with 1 indicating perfect classification performance and 0.5 indicating random chance.

## 8 SELECTED MODELS AND HYPERPARAMETERS

Table S4 displays the selected models and hyperparameters in each training fold or repetition (the inner layer of a nested cross-validation model) using random-split, time-split, participant-split, and personalized cross-validation strategies. Hyperparameters were found using a gridsearch with the following possible values: NN Hidden layer sizes $\in$ {[200], [100], [50], [200, 100], [100, 50], [200, 100, 50]}, L2 regularization $\in$ $\{10^{-6}, 10^{-5}, 10^{-4}, 10^{-3}, 10^{-2}\}$, NN activation function $\in$ {logistic, (log), tanh, relu}, learning rate $\in$ {0.01, 0.1}, RF % sampled features $\in$ {0.1, 0.2, 0.3, 0.4, 0.5}, RF minimum leaf size $\in$ {1, 2, 3, 4, 5}, and RF # estimators $\in$ {100, 200, 500}.

In the table, the hyperparameters for random forest are presented as RF (Percentage of sampled features, minimum leaf size, number of estimators). Percentage of sampled features refers to the percentage of features used to make each split. The minimum leaf size is the minimum number of observations required in a leaf node. Number of estimators refers to the number of trees grown in the forest.

For mixed effect random forest (MERF), the hyperparameters were fixed as the best performing RF parameter values. Thus, they are presented as MERF(Percentage of sampled features, minimum leaf size, number of estimators).

For Feedforward Neural Network (NN) classifier, the hyperparameters are presented as NN(nonlinear activation function, weight of the L2 regularization, number of and size of hidden layers, initial learning rate for the ADAM optimizer) in the table.

## 9 ROC-AUC FOR RANDOM-SPLIT AND TIME-SPLIT CROSS-VALIDATION

Figure 3a shows the ROC of all test folds in the outer layer of the random-split cross-validation. Figure 3b shows the ROC of all test folds in the outer layer of the time-split cross-validation. The dashed line indicates a random classifier. The closer the curve is to the upper left corner the better the performance of the classifier. A true positive rate of 1.0 and a false positive rate of 0.0 would be a perfect classifier. As the performance measures of the participant-based cross-validation were all lower than the random cross-validation, no ROC curves were generated for the participant-based cross-validation.

## 10 SHAP-VALUES FOR THE MOST IMPORTANT FEATURES

Figure S4 shows the SHAP values for the participant-split or -based cross-validation model. As the participant-split model selected mixed effects random forest (MERF) three times, NN three times, and RF twice, the SHAP values were derived from these models. These SHAP values seem to show more directionality than those from the random-split model presented in the main text. For example, low values for the frequency domain feature, the real component of the interquartile range of the BVP (BVP_freq_iqr_real) seem to influence the model to classify events as OCD-events. The inverse seems to be true for low values of the (BVP_freq_max_real). Although neither case shows a clear distintiction.

Figure S5 shows the SHAP values for the time-split or temporal cross-validation model. These SHAP values were derived from MERF and RF models selected in the inner layer. The same features that ranked as important in the random-split and participant-split models ranked as important for the time-split model. Here, low and high feature values had the same probability of resulting in a OCD-event or nonevent classification.

## 11 COMPARISON OF FEATURES UNDER CONDITIONS OF REST AND EXPOSURE

To explore main, linear effects, we created box plots of all $66$ features under controlled conditions of rest and exposure in the lab (see Figure S6). The main and linear effects need to be interpreted with caution due to the small sample size. We were also interested to see if important features in classification decisions were differed in controlled conditions of OCD-events (exposure) and rest. The following HR features were among the top $20$ most important features in classifying OCD-events and nonevents (see Figure 11: standard deviation (HR_std), the $25\%$ quantile (HR_25quantile), and maximum HR (HR_max). The box plots of these features did not reveal differences in the expected directions. All three features had higher values in the rest condition than in the exposure condition. These results are consistent with group and individual graphs of the whole HR signal during rest and exposure (Figures S8 and S9) that show that participants had higher heart rate during the resting period than during the exposure condition. This is contrary to expectations (see Table S5), but may be due to sampling only the first five minutes after exposure start. Other periods of the exposure condition may be more arousing, such as the exposure

preparation stage, in which the individual anticipates the averse stimulus or later in the exposure condition when an exposure may be intensified.

One skin temperature feature was among the top $20$ most important features in classifying OCD-events and nonevents (see Figure 11: maximum temperature (TEMP_max; see Figure S6). The box plot comparing TEMP_max in the rest and exposure conditions show no difference. However, the standard deviation and slope of skin temperature show more variation in the exposure condition than the resting period with higher standard deviation values and lower slope values in the exposure condition. This last result suggests that the rate of change in skin temperature was slower in the exposure condition.

Two EDA features appeared in the top $20$ most important features in classifying OCD-events and nonevents (see Figure 11: phasic components with power in the ultrahigh frequency band (Phasic_BandPower_0.4_1.0) and the low frequency band (Phasic_BandPower_0.04_0.15). The box plot comparing these phasic EDA features in the rest and exposure conditions show no difference difference in Phasic_BandPower_0.04_0.15 but more variation in Phasic_BandPower_0.4_1.0 in the resting period than during exposure (see Figure 10 in the main text). Other differences are are also notable in features related to the phasic component, i.e., skin conductance response, and the tonic component, i.e., skin conductance level.

## 12 POWER ANALYSIS

We investigated the amount of training data and tagged OCD events needed to train classification models capable of detecting in-the-wild OCD events. Figure S10 displays the predictive performance of models trained using two-layer random 10-fold cross-validation. Data from participants with many observations have been randomly down-sampled to match the amount from participants with fewer observations. Reduction of the training data had little effect on the accuracy but the precision and recall decreased with less training data. We also calculated p-values for the hypothesis of equal ROC-AUC between down-sampled models and models with no down-sampling (Hanley and McNeil, 1982). We down-sampled to the participant with the least observations, $< 10^{-4}$, the participant with median number of observations, $1.2 \cdot 10^{-4}$, and to next-highest, $7 \cdot 10^{-4}$. Based on these tests, we can reject the hypothesis of equal ROC-AUC between down-sampled models and models with no down-sampling for every level of down-sampling. This indicates that reduction of the training data through random down-sampling reduces the power of the detection.

To estimate the amount training data needed to achieve a certain level of performance, we fit an inverse power law to the measured performance metrics as a function of the number of observations (Figueroa, Zeng-Treitler, Kandula, and Ngo, 2012). Figure S11 illustrates that the accuracy has already reached its asymptotic value using all our recorded data. Thus, more training will not improve the accuracy. However, obtaining a recall of $70\%$ would require an estimated $40.000$ training points assuming the distribution between participants and classes remain similar. The recall grows asymptotically towards approximately $75\%$ meaning that the estimated best case model may only detect $75\%$ of cases. However, the ROC-AUC increases asymptotically towards approximately $90\%$ indicating that the recall may further improve by adjusting the detection threshold as discussed in Section 3.2.1 of the main text. The extrapolated increase of F1-score, precision, recall, and ROC-AUC is mutually exclusive with the asymptotic behavior of the accuracy. For the extrapolated increase to happen, the accuracy would need to increase further. Similarly, for the asymptotic behavior of the accuracy to happen, the remaining performance measures would be required to similarly level off. Thus, we cannot conclusively estimate the training set size required to improve

the power of the classification algorithm based on these results. To ensure the down-sampled training data contained as many OCD-events from every participant as possible, we chose to down-sample high observation participants (participants with many OCD-events) instead of selecting observations randomly. This down-sampling caused changes in the participant distribution, which would have a great impact on the recall if the down-sampling of high observation participants resulted in removing of easily identifiable OCD-events. This might indicate that the recall is more likely to level off.

## 13 TABLES

**Table S1.** Obsessive compulsive disorder (OCD) symptom types, counts and severity at T1

| Participant | Symmetry | Forbidden Thoughts | Cleaning | Hoarding | Total Symptom Count | |
|---|---|---|---|---|---|---|
| 0 | 3 | 1 | 6 | 0 | 10 | |
| 1 | 1 | 6 | 6 | 0 | 13 | |
| 2 | 4 | 2 | 0 | 2 | 8 | |
| 3 | 2 | 3 | 0 | 0 | 5 | |
| 4 | 0 | 0 | 1 | 0 | 1 | |
| 5 | 6 | 8 | 4 | 2 | 20 | |
| 6 | 0 | 1 | 2 | 1 | 4 | |
| 7 | 6 | 4 | 2 | 1 | 13 | |
| 8 | 3 | 3 | 0 | 0 | 6 | |
| Participant | Symptom Severity | Time | Interference | Distress | Resistance | Control |
| 0 | 27 | 7 | 4 | 4 | 6 | 6 |
| 1 | 27 | 5 | 5 | 5 | 6 | 6 |
| 2 | 29 | 6 | 4 | 6 | 6 | 7 |
| 3 | 23 | 3 | 5 | 5 | 6 | 4 |
| 4 | 11 | 2 | 1 | 2 | 3 | 3 |
| 5 | 24 | 4 | 3 | 6 | 5 | 6 |
| 6 | 27 | 5 | 3 | 6 | 7 | 6 |
| 7 | 28 | 8 | 4 | 4 | 6 | 6 |
| 8 | 25 | 6 | 4 | 4 | 5 | 6 |

**Table S2.** Spearman correlations between symptom counts and severity and number of recorded hour and tagged events. (N=9)

| | Age | Symmetry | Forbidden Thoughts | Cleaning | Hoarding | Total Symptom Count | Symptom Severity | |
|---|---|---|---|---|---|---|---|---|
| Age | - | 0.65 (0.06) | 0.49 (0.18) | -0.08 (0.84) | 0.50 (0.17) | 0.47 (0.21) | 0.39 (0.29) | |
| Symmetry | 0.65 (0.06) | - | 0.58 (0.10) | 0.01 (0.97) | 0.57 (0.11) | 0.75 (0.02) | 0.39 (0.29) | |
| Forbidden Thoughts | 0.49 (0.18) | 0.58 (0.10) | - | 0.23 (0.55) | 0.27 (0.48) | 0.81 (0.01) | 0.10 (0.79) | |
| Cleaning | -0.08 (0.84) | 0.01 (0.97) | 0.23 (0.55) | - | -0.04 (0.92) | 0.57 (0.11) | 0.16 (0.69) | |
| Hoarding | 0.50 (0.17) | 0.57 (0.11) | 0.27 (0.48) | -0.04 (0.92) | - | 0.38 (0.32) | 0.47 (0.20) | |
| Total Symptom Count | 0.47 (0.21) | 0.75 (0.02) | 0.81 (0.01) | 0.57 (0.11) | 0.38 (0.32) | - | 0.39 (0.30) | |
| Symptom Severity | 0.39 (0.29) | 0.39 (0.29) | 0.10 (0.79) | 0.16 (0.69) | 0.47 (0.20) | 0.39 (0.30) | - | |
| Time | 0.56 (0.12) | 0.54 (0.13) | 0.10 (0.79) | 0.18 (0.64) | 0.18 (0.65) | 0.43 (0.24) | 0.81 (0.01) | |
| Interference | -0.08 (0.84) | 0.12 (0.76) | 0.43 (0.25) | -0.03 (0.94) | -0.35 (0.36) | 0.29 (0.45) | 0.27 (0.49) | |
| Distress | 0.25 (0.52) | 0.17 (0.67) | 0.34 (0.38) | 0.01 (0.97) | 0.70 (0.04) | 0.25 (0.52) | 0.32 (0.41) | |
| Resistance | -0.02 (0.95) | -0.13 (0.74) | -0.06 (0.88) | 0.19 (0.63) | 0.19 (0.63) | 0.01 (0.98) | 0.63 (0.07) | |
| Control | 0.61 (0.08) | 0.53 (0.15) | 0.25 (0.51) | 0.09 (0.82) | 0.62 (0.08) | 0.50 (0.17) | 0.86 (0.00) | |
| Days Worn | 0.34 (0.37) | -0.07 (0.85) | -0.13 (0.74) | 0.34 (0.36) | -0.16 (0.68) | -0.00 (0.99) | 0.23 (0.55) | |
| Hours per day | 0.10 (0.80) | -0.22 (0.57) | 0.18 (0.65) | 0.13 (0.74) | -0.45 (0.22) | -0.01 (0.98) | 0.00 (1.00) | |
| Tags per Hour | 0.53 (0.14) | 0.19 (0.62) | 0.71 (0.03) | 0.15 (0.69) | -0.24 (0.53) | 0.43 (0.25) | -0.07 (0.86) | |
| | Time | Interference | Distress | Resistance | Control | Days Worn | Hours per day | Tags per Hour |
| Age | 0.56 (0.12) | -0.08 (0.84) | 0.25 (0.52) | -0.02 (0.95) | 0.61 (0.08) | 0.34 (0.37) | 0.10 (0.80) | 0.53 (0.14) |
| Symmetry | 0.54 (0.13) | 0.12 (0.76) | 0.17 (0.67) | -0.13 (0.74) | 0.53 (0.15) | -0.07 (0.85) | -0.22 (0.57) | 0.19 (0.62) |
| Forbidden Thoughts | 0.10 (0.79) | 0.43 (0.25) | 0.34 (0.38) | -0.06 (0.88) | 0.25 (0.51) | -0.13 (0.74) | 0.18 (0.65) | 0.71 (0.03) |
| Cleaning | 0.18 (0.64) | -0.03 (0.94) | 0.01 (0.97) | 0.19 (0.63) | 0.09 (0.82) | 0.34 (0.36) | 0.13 (0.74) | 0.15 (0.69) |
| Hoarding | 0.18 (0.65) | -0.35 (0.36) | 0.70 (0.04) | 0.19 (0.63) | 0.62 (0.08) | -0.16 (0.68) | -0.45 (0.22) | -0.24 (0.53) |
| Total Symptom Count | 0.43 (0.24) | 0.29 (0.45) | 0.25 (0.52) | 0.01 (0.98) | 0.50 (0.17) | -0.00 (0.99) | -0.01 (0.98) | 0.43 (0.25) |
| Symptom Severity | 0.81 (0.01) | 0.27 (0.49) | 0.32 (0.41) | 0.63 (0.07) | 0.86 (0.00) | 0.23 (0.55) | 0.00 (1.00) | -0.07 (0.86) |
| Time | - | 0.27 (0.49) | -0.10 (0.80) | 0.37 (0.32) | 0.67 (0.05) | 0.52 (0.15) | 0.18 (0.65) | 0.18 (0.63) |
| Interference | 0.27 (0.49) | - | 0.05 (0.89) | 0.38 (0.32) | 0.16 (0.69) | -0.20 (0.61) | 0.18 (0.63) | 0.40 (0.29) |
| Distress | -0.10 (0.80) | 0.05 (0.89) | - | 0.49 (0.18) | 0.55 (0.12) | -0.40 (0.29) | -0.55 (0.12) | -0.11 (0.77) |
| Resistance | 0.37 (0.32) | 0.38 (0.32) | 0.49 (0.18) | - | 0.39 (0.30) | 0.25 (0.52) | -0.03 (0.94) | -0.10 (0.80) |
| Control | 0.67 (0.05) | 0.16 (0.69) | 0.55 (0.12) | 0.39 (0.30) | - | -0.02 (0.95) | -0.30 (0.44) | -0.01 (0.98) |
| Days Worn | 0.52 (0.15) | -0.20 (0.61) | -0.40 (0.29) | 0.25 (0.52) | -0.02 (0.95) | - | 0.66 (0.06) | 0.37 (0.33) |
| Hours per day | 0.18 (0.65) | 0.18 (0.63) | -0.55 (0.12) | -0.03 (0.94) | -0.30 (0.44) | 0.66 (0.06) | - | 0.65 (0.06) |
| Tags per Hour | 0.18 (0.63) | 0.40 (0.29) | -0.11 (0.77) | -0.10 (0.80) | -0.01 (0.98) | 0.37 (0.33) | 0.65 (0.06) | - |

Table S3: All features calculated from measured physiological signals.

| | Feature | Description | Section |
|---|---|---|---|
| | | **Blood volume pulse (BVP)/PPG signal** | |
| 1 | BVP_RR | Average interbeat interval/ R-R interval | Time-domain |
| 2 | BVP_RMSSD | root mean square of successive differences | |
| 3 | BVP_time_mean | Mean | |
| 4 | BVP_time_std | Standard deviation | |
| 5 | BVP_time_median | Median | |
| 6 | BVP_time_min | Minimum | |
| 7 | BVP_time_max | Maximum | |
| 8 | BVP_slope | Slope | |
| 9 | BVP_freq_mean_real | Mean for real component | Frequency domain |
| 10 | BVP_freq_std_real | Standard deviation for real component | |
| 11 | BVP_freq_median_real | Median for real component | |
| 12 | BVP_freq_min_real | Minimum for real component | |
| 13 | BVP_freq_max_real | Maximum for real component | |
| 14 | BVP_freq_iqr_real | Interquartile range for real component | |
| 15 | BVP_freq_sma_real | Sum of frequencies for real component | |
| 16 | BVP_freq_mean_im | Mean for imaginary component | |
| 17 | BVP_freq_std_im | Standard deviation for imaginary component | |
| 18 | BVP_freq_median_im | Median for imaginary component | |
| 19 | BVP_freq_min_im | Minimum for imaginary component | |
| 20 | BVP_freq_max_im | Maximum for imaginary component | |
| 21 | BVP_freq_iqr_im | Interquartile range for imaginary component | |
| 22 | BVP_freq_sma_im | Sum of frequencies for imaginary component | |
| 23 | BVP_min_Slope | Minimum slope | Shape |
| 24 | BVP_max_Slope | Maximum slope | |
| | | **Heart rate (HR)** | |
| 25 | HR_mean | Mean | |
| 26 | HR_std | Standard deviation | |
| 27 | HR_25quantile | 25% quantlie | |
| 28 | HR_Mmedian | Median | |
| 29 | HR_75quantile | 75% quantile | |
| 30 | HR_min | Minimum | |
| 31 | HR_max | Maximum | |
| 32 | HR_quantiledeviation | Interquartile range | |
| 33 | HR_slope | Slope | |
| | | **Skin temperature** | |
| 34 | TEMP_mean | Mean | |
| 35 | TEMP_std | Standard deviation | |
| 36 | TEMP_min | Minimum | |
| 37 | TEMP_max | Maximum | |
| 38 | TEMP_slope | Slope | |

| | | | |
|---|---|---|---|
| | | **EDA** | |
| 39 | Tonic_25quantile | 25% quantlie | Tonic component |
| 40 | Tonic_median | Median | |
| 41 | Tonic_75quantile | 75% quantile | |
| 42 | Tonic_quantiledeviation | Interquartile range | |
| 43 | Tonic_min | Minimum | |
| 44 | Tonic_max | Maximum | |
| 45 | Tonic_slope | Slope | |
| 46 | Phasic_mean | Mean | Phasic component |
| 47 | Phasic_std | Standard deviation | |
| 48 | Phasic_numPeaks | Number of peaks | |
| 49 | Phasic_avgPeakAmplitude | Average peak amplitude | |
| 50 | Phasic_avgPeakResponseTime | Average peak response time | |
| 51 | Phasic_BandPower_0.01_0.04 | Power in the ultralow frequency band (0.01-0.04Hz) | |
| 52 | Phasic_BandPower_0.04_0.15 | Power in the low frequency band (0.04-0.15Hz) | |
| 53 | Phasic_BandPower_0.15_0.4 | Power in the high frequency band (0.15-0.4Hz) | |
| 54 | Phasic_BandPower_0.4_1.0 | Power in the ultrahigh frequency band (0.4-1.0Hz) | |
| 55 | EDA_mean | Mean | Unnormalized |
| 56 | EDA_std | Standard deviation | |
| 57 | EDA_25quantile | 25% quantlie | |
| 58 | EDA_median | Median | |
| 59 | EDA_75quantile | 75% quantile | |
| 60 | EDA_quantiledeviation | Interquartile range | |
| 61 | EDA_min | Minimum | |
| 62 | EDA_max | Maximum | |
| 63 | EDA_BandPower_0.01_0.04 | Power in the ultralow frequency band (0.01-0.04Hz) | |
| 64 | EDA_BandPower_0.04_0.15 | Power in the low frequency band (0.04-0.15Hz) | |
| 65 | EDA_BandPower_0.15_0.4 | Power in the high frequency band (0.15-0.4Hz) | |
| 66 | EDA_BandPower_0.4_1.0 | Power in the ultrahigh frequency band (0.4-1.0Hz) | |

**Table S4.** The selected model and hyperparameters in each training fold/repetition using different cross-validation strategies.

| CV-type | Fold/Repetion 1 | Fold/Repetion 2 | Fold/Repetion 3 | Fold/Repetion 4 | Fold/Repetion 5 | Fold/Repetion 6 | Fold/Repetion 7 | Fold/Repetion 8 | Fold/Repetion 9 | Fold/Repetion 10 |
|---|---|---|---|---|---|---|---|---|---|---|
| Random | MERF(0.1, 1, 500) | MERF(0.1, 1, 200) | MERF(0.1, 3, 500) | MERF(0.1, 1, 500) | MERF(0.1, 2, 200) | MERF(0.2, 1, 500) | MERF(0.1, 1, 500) | MERF(0.1, 3, 100) | MERF(0.1, 2, 500) | MERF(0.1, 2, 500) |
| Temporal | MERF(0.3, 1, 200) | MERF(0.4, 2, 100) | MERF(0.4, 1, 100) | MERF(0.3, 5, 500) | MERF(0.4, 5, 500) | MERF(0.4, 3, 500) | MERF(0.2, 4, 200) | RF(0.1, 3, 100) | MERF(0.4, 4, 500) | RF(0.3, 2, 200) |
| Participant | MERF(0.4, 2, 200) | MERF(0.3, 1, 100) | NN(log., 1e-06, [200, 100], 0.1) | NN(relu, 0.0001, [200, 100, 50], 0.1) | NN(log., 0.0001, [200, 100], 0.1) | MERF(0.2, 1, 100) | RF (0.2, 1, 200) | RF (0.1, 1, 100) | | |
| Personal ID 1 | NN(log., 1e-06, [100, 50], 0.01) | NN(relu, 0.01, [50], 0.1) | NN(log., 0.0001, [100, 50], 0.1) | NN(log., 1e-05, [100], 0.1) | NN(log., 1e-06, [100, 50], 0.1) | NN(log., 1e-06, [200], 0.1) | NN(log., 1e-06, [200, 100], 0.01) | NN(tanh, 0.0001, [100], 0.1) | NN(log., 1e-06, [50], 0.1) | NN(log., 1e-05, [200, 100], 0.01) |
| Personal ID 2 | RF (0.3, 4, 200) | NN(log., 1e-06, [200, 100], 0.01) | NN(log., 0.01, [100], 0.01) | RF (0.4, 3, 200) | NN(log., 1e-05, [200, 100, 50], 0.01) | NN(log., 0.0001, [100], 0.1) | NN(log., 0.001, [200, 100], 0.001) | NN(log., 1e-05, [200], 0.001) | NN(log., 0.01, [200], 0.01) | NN(log., 0.01, [100], 0.01) |
| Personal ID 3 | NN(log., 1e-06, [100, 50], 0.1) | NN(tanh, 0.001, [200], 0.1) | NN(log., 1e-06, [200], 0.1) | NN(relu, 0.001, [200, 100], 0.1) | NN(relu, 1e-06, [100], 0.1) | NN(tanh, 0.0001, [50], 0.1) | NN(log., 0.001, [200], 0.1) | NN(relu, 0.01, [100, 50], 0.1) | NN(tanh, 1e-06, [200, 100, 50], 0.1) | NN(relu, 1e-06, [200, 100], 0.1) |
| Personal ID 4 | NN(log., 1e-06, [200], 0.1) | NN(log., 1e-06, [200], 0.01) | NN(log., 1e-06, [200], 0.01) | NN(log., 1e-06, [200], 0.01) | NN(log., 1e-06, [200], 0.01) | NN(log., 1e-06, [200], 0.001) | NN(log., 1e-06, [200], 0.001) | NN(log., 1e-06, [200], 0.001) | NN(log., 1e-06, [200], 0.01) | NN(log., 1e-06, [200], 0.01) |
| Personal ID 5 | NN(log., 0.01, [200, 100], 0.1) | NN(log., 1e-06, [100, 50], 0.1) | RF(0.1, 1, 500) | RF(0.1, 1, 100) | NN(log., 1e-06, [200, 100], 0.1) | RF(0.1, 1, 100) | RF(0.1, 3, 200) | NN(tanh, 0.001, [200, 100, 50], 0.1) | NN(log., 1e-06, [200, 100, 50], 0.001) | RF(0.1, 1, 100) |
| Personal ID 6 | RF(0.1, 3, 500) | NN(log., 1e-06, [100], 0.1) | NN(log., 1e-06, [100, 50], 0.1) | NN(relu, 0.0001, [50], 0.1) | NN(log., 1e-06, [100, 50], 0.1) | NN(log., 0.001, [100], 0.1) | NN(tanh, 0.0001, [100, 50], 0.1) | NN(log., 1e-05, [100, 50], 0.1) | NN(log., 0.0001, [200], 0.1) | NN(log., 0.0001, [50], 0.1) |
| Personal ID 7 | NN(log., 0.01, [50], 0.1) | NN(relu, 0.001, [100], 0.1) | NN(tanh, 0.001, [50], 0.01) | NN(relu, 1e-05, [200, 100, 50], 0.001) | NN(log., 0.001, [100, 50], 0.1) | NN(relu, 0.001, [200, 100], 0.01) | NN(log., 0.01, [100, 50], 0.1) | NN(tanh, 0.0001, [50], 0.1) | NN(log., 0.001, [200, 100, 50], 0.1) | NN(relu, 0.001, [200, 100, 50], 0.001) |
| Personal ID 8 | RF(0.3, 1, 500) | RF(0.2, 3, 100) | RF(0.2, 2, 500) | RF(0.3, 2, 500) | RF(0.4, 5, 500) | RF(0.2, 2, 200) | RF(0.2, 4, 500) | RF(0.4, 3, 200) | RF(0.3, 2, 100) | RF(0.4, 3, 100) |

**Table S5.** Physiological responses to obsessive-compulsive disorder (OCD)-events (mental states). The OCD event-lab refers to our experient of comparing physiological signals during conditions of rest and exposure. Physiological responses to mental states are based on the literature (Brown et al., 2012; Collet et al., 1997; Gross et al., 1994; Mohammed et al., 2021). EDA: electrodermal activity. SCL: skin conductance level. SCR: skin conductance response.

| Mental states within OCD | Heart rate | Tonic EDA (SCL) | Phasic EDA (SCR) | Skin temperature |
|---|---|---|---|---|
| Fear (immediate threat)/ Anxiety (anticipated threat; agitation) | Increased | Increased | Increased | Decreased |
| Anger (frustration) | Increased | Increased | Increased | Decreased |
| Disgust (contamination) | Increased | Increased | Increased | Decreased |
| Disgust (mutilation) | Decreased | Increased | Increased | No information |
| Sadness (noncrying, acute) | Decreased | Decreased | Increased | Decreased |
| Sadness (crying; anticipated) | Increased | Increased | No information | Decreased |
| Embarrassment (shame, social anxiety, rejection) | Increased | Increased | No information | No information |
| Cognitive effort associated with monitoring, suppression, uncertainty | Increased | Increased | No change | No information |
| OCD event - lab | Increased | unclear | Increased | No difference |

## REFERENCES


Bloch, M. H., Landeros-Weisenberger, A., Rosario, M. C., Pittenger, C., and Leckman, J. F. (2008). Meta-analysis of the symptom structure of obsessive-compulsive disorder. *American Journal of Psychiatry* 165, 1532–1542

Brown, R., James, C., Henderson, L. A., and Macefield, V. G. (2012). Autonomic markers of emotional processing: skin sympathetic nerve activity in humans during exposure to emotionally charged images. *Frontiers in physiology* 3

Collet, C., Vernet-Maury, E., Delhomme, G., and Dittmar, A. (1997). Autonomic nervous system response patterns specificity to basic emotions. *Journal of the autonomic nervous system* 62, 45–57

Figueroa, R. L., Zeng-Treitler, Q., Kandula, S., and Ngo, L. H. (2012). Predicting sample size required for classification performance. *BMC medical informatics and decision making* 12, 8

[Dataset] Fromberg, L., Das, S., and Clemmensen, L. K. H. (2023). Pre-processing blood-volume-pulse for in-the-wild applications. doi:10.48550/arXiv.2304.14186

Goodman, W., Price, L., Rasmussen, S., Riddle, M., and Rapoport, J. (1991). Children's yale-brown obsessive compulsive scale (cy-bocs). *New Haven, Connecticut: Clinical Neuroscience Unit* 29, 31–51

Gross, J. J., Fredrickson, B. L., and Levenson, R. W. (1994). The psychophysiology of crying. *Psychophysiology* 31, 460–468

Hanley, J. A. and McNeil, B. J. (1982). The meaning and use of the area under a receiver operating characteristic (roc) curve. *Radiology* 143, 29–36. doi:https://doi.org/10.1148/radiology.143.1.7063747

Krishnan, R., Natarajan, B., and Warren, S. (2010). Two-stage approach for detection and reduction of motion artifacts in photoplethysmographic data. *IEEE Transactions on Biomedical Engineering* 57, 1867–1876. doi:10.1109/TBME.2009.2039568

Leckman, J. F., Grice, D. E., Boardman, J., Zhang, H., Vitale, A., Bondi, C., et al. (1997). Symptoms of obsessive-compulsive disorder. *American Journal of Psychiatry* 154, 911–917

Makowski, D., Pham, T., Lau, Z. J., Brammer, J. C., Lespinasse, F., Pham, H., et al. (2021). Neurokit2: A python toolbox for neurophysiological signal processing. *Behavior Research Methods* 53, 1689–1696. doi:10.3758/s13428-020-01516-y

Mohammed, A.-R., Kosonogov, V., and Lyusin, D. (2021). Expressive suppression versus cognitive reappraisal: effects on self-report and peripheral psychophysiology. *International Journal of Psychophysiology* 167, 30–37

Olesen, K. V., Lønfeldt, N. N., Das, S., Pagsberg, A. K., and Clemmensen, L. K. H. (2023). Feasibility of predicting obsessive-compulsive disorder events in children and adolescents from biosignals in-the-wild - a wrist angel analysis plan. *JMIR Preprints 48571* doi:10.2196/preprints.48571

# 14 FIGURES

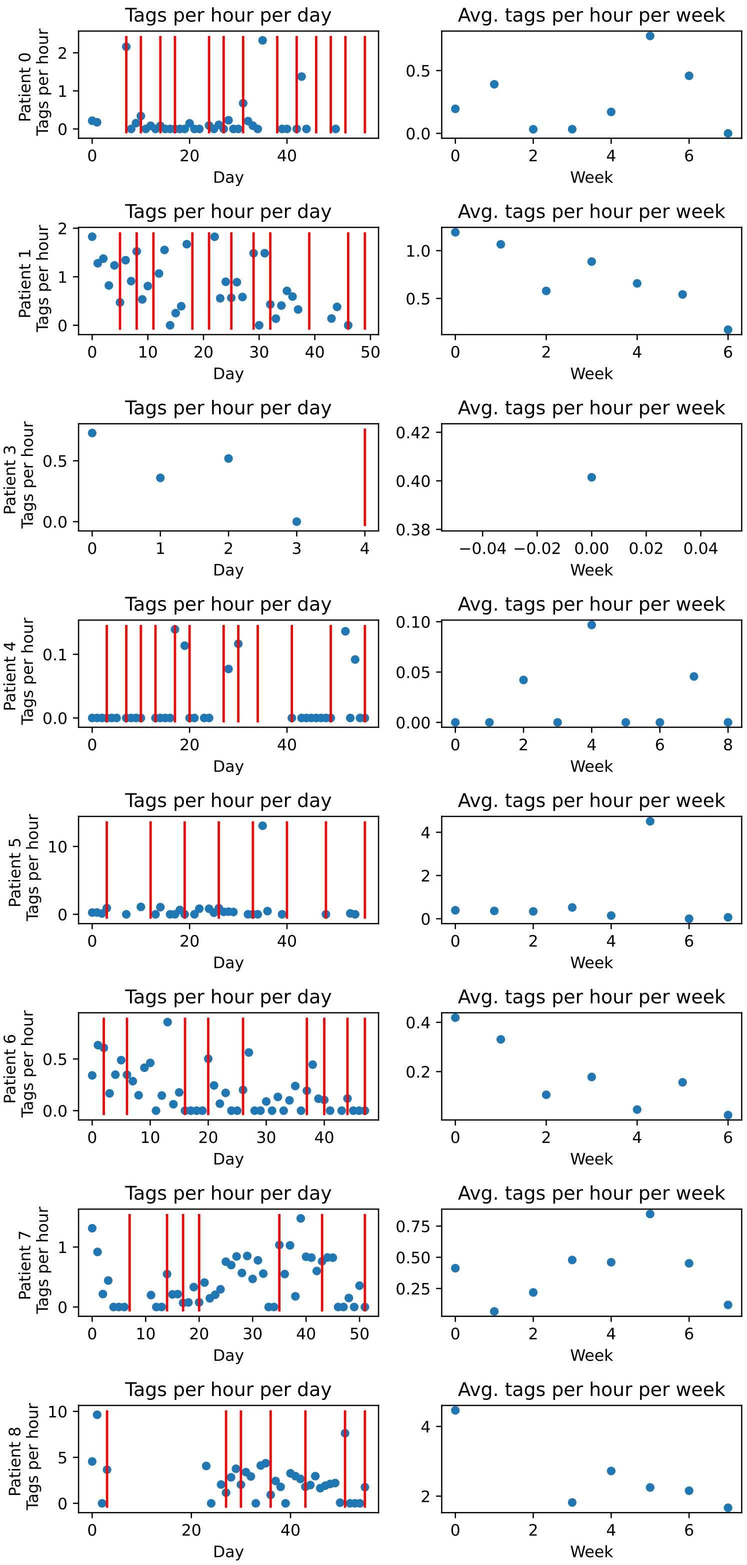


**Figure S1.** Left: Number of tagged OCD events per hour split by participant and day. Red lines denote the days on which researchers met with participants. Right: Average number of tagged OCD events per hour split by participant and week.

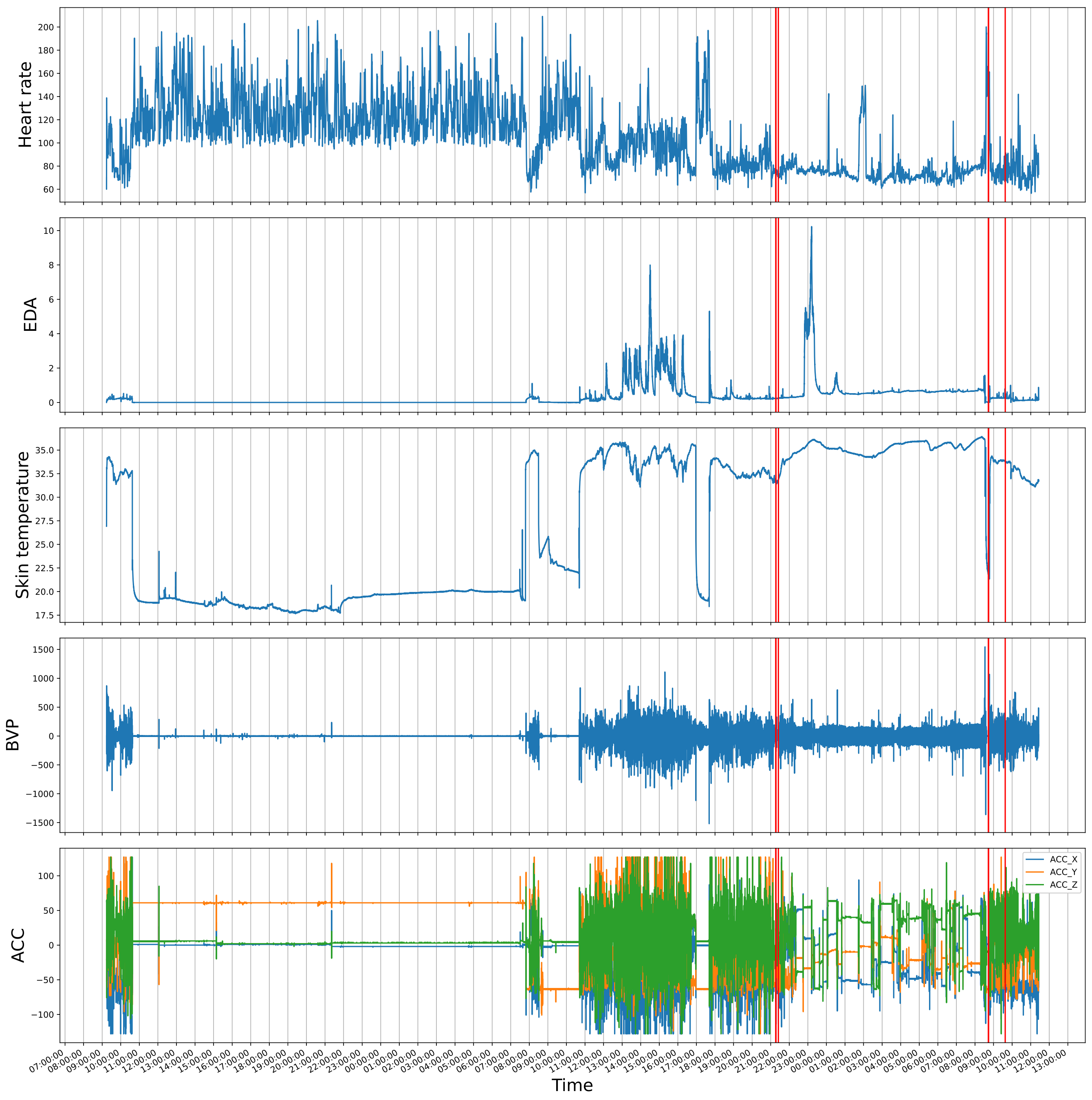


**Figure S2.** Recording from a wristband containing both a period of identified sleep and periods when the wristband was not worn. Red lines denote tagged OCD events.

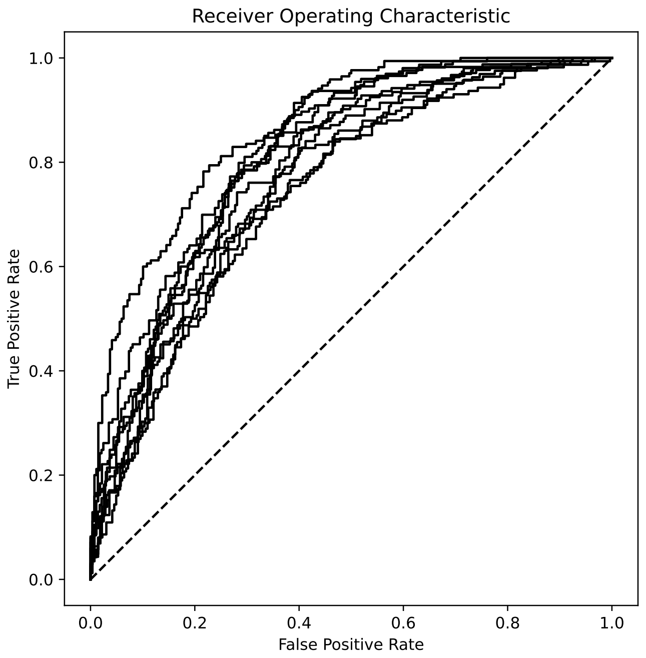


**Figure 3a.** Random cross-validation.

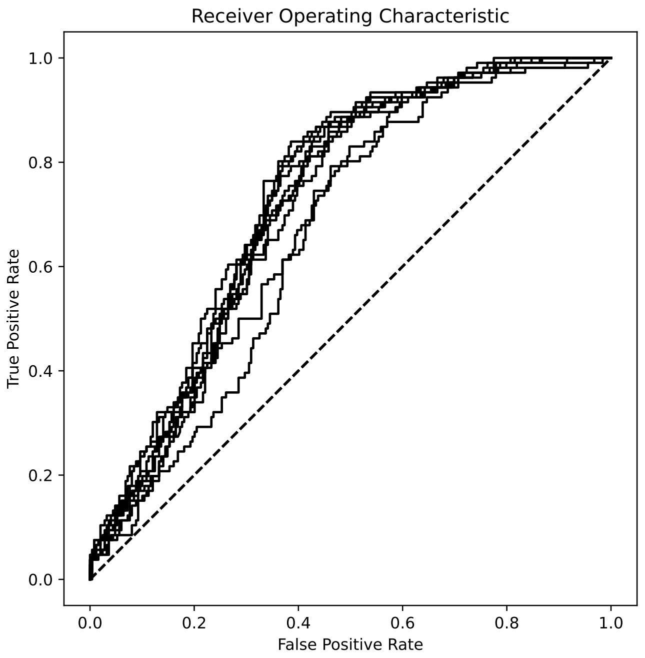


**Figure 3b.** Temporal cross-validation.

**Figure 3.** The receiver operating characteristic of all outer test folds using random 10-fold (a) and temporal (b) cross-validation.

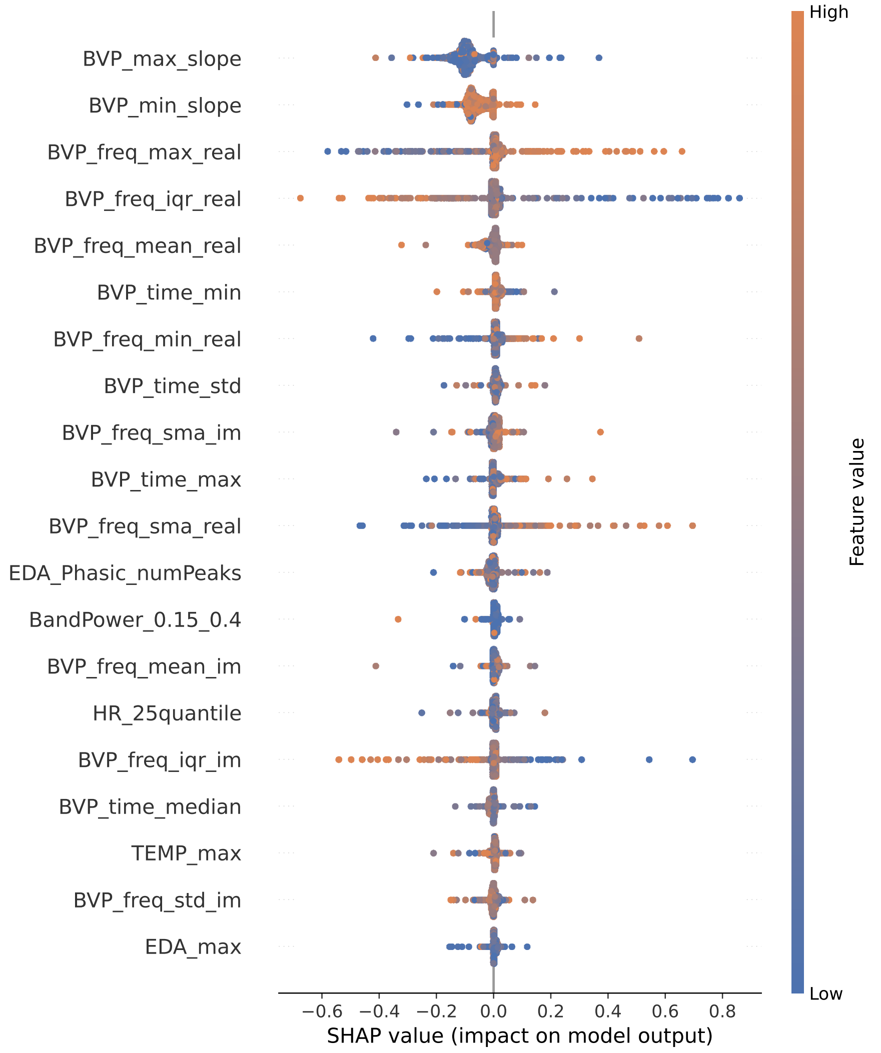


**Figure S4.** SHAP-values for the most important features using participant-based cross-validation. For participant-based models, mixed effects random forest (MERF) was selected three times, feedforward neural network (NN) was selected three times, and random forest (RF) was selected twice. Note, there was no overlap between folds here. Test cases were evaluated with different models.

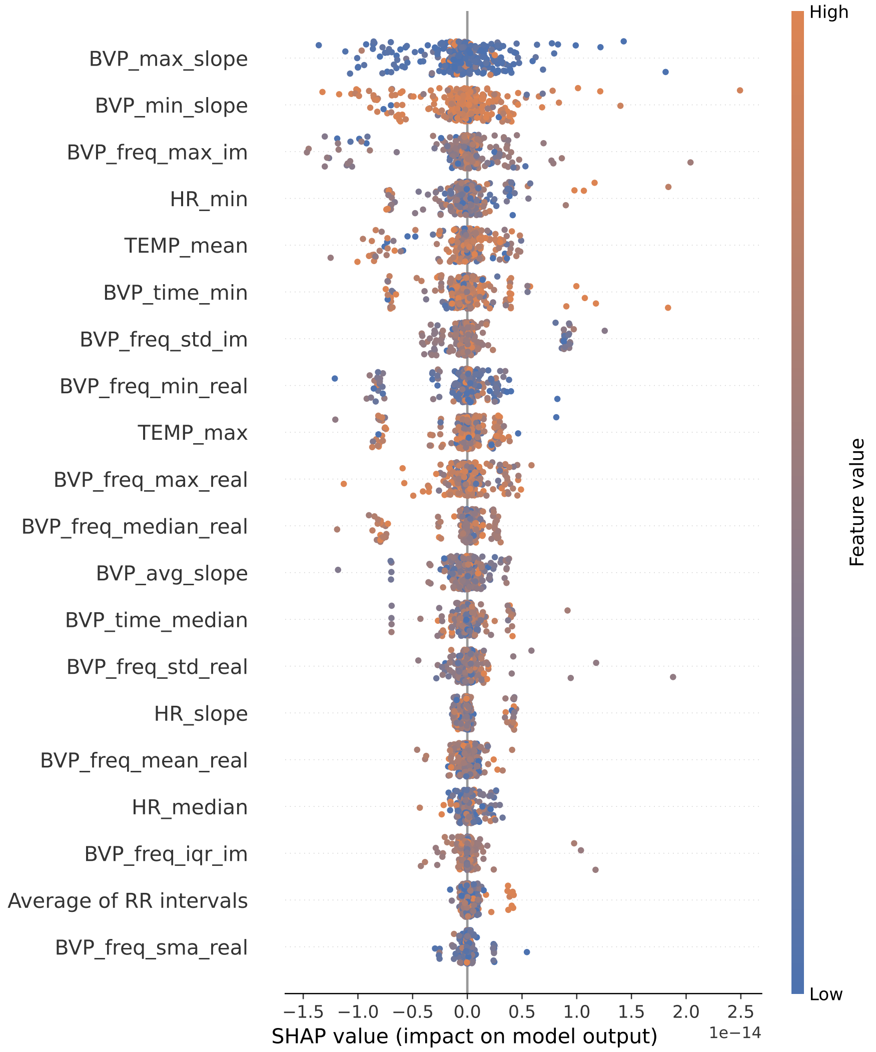


**Figure S5.** SHAP-values for the most important features using temporal cross-validation. For Temporal models, MERF was selected 8 times, RF 2 times. Here we have 10 repetitions of the same test cases. So SHAP values are averages of these 10 repetitions.

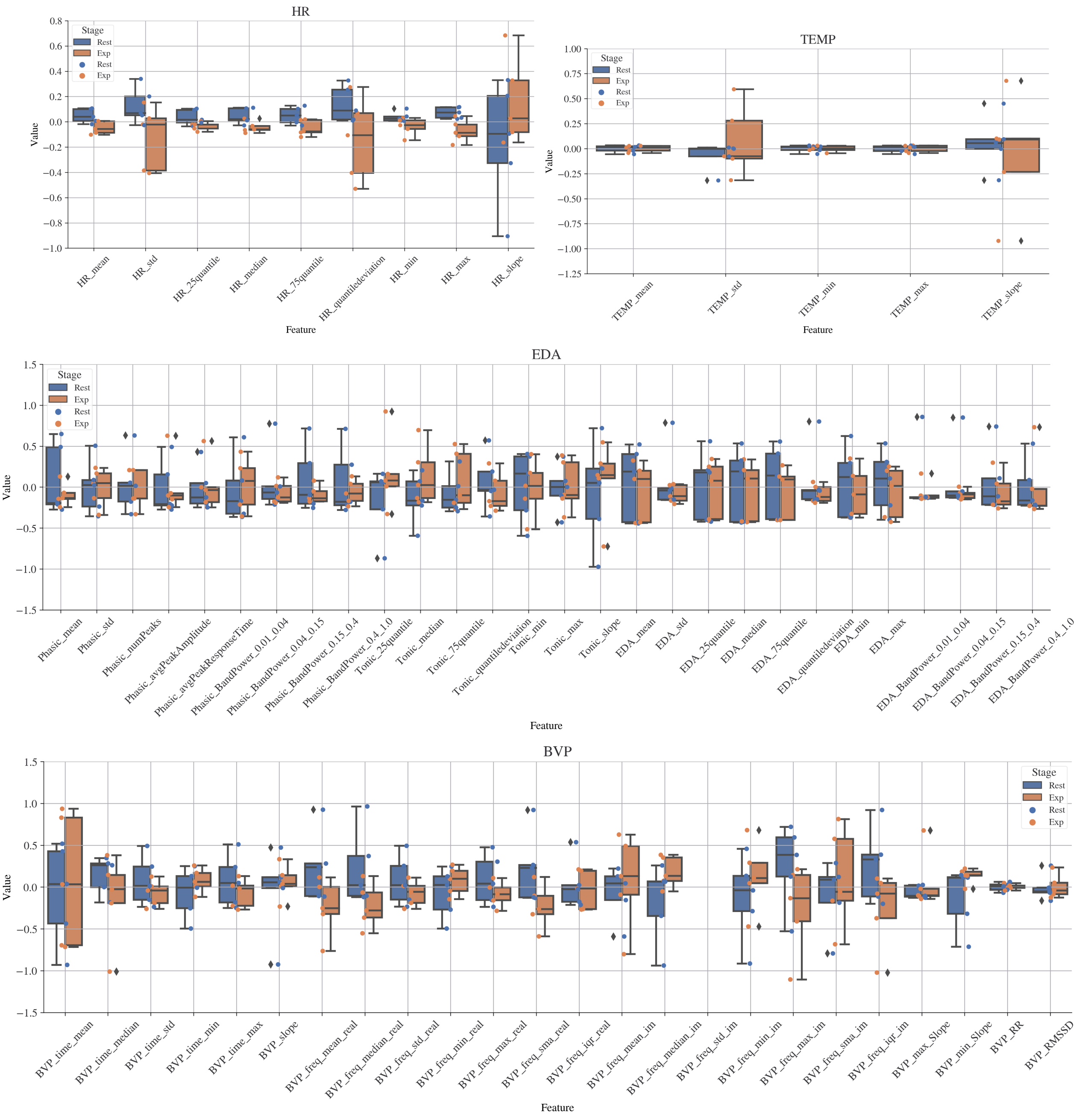


**Figure S6.** box plot of the features extracted from the heart-rate (HR), electrodermal activity (EDA), temperature (TEMP) and blood-volume pulse (BVP) signals during the conditions of rest and exposure (n=5) at Time 1. Only includes participants whose time stamps in the E4 data could be synchronized with the start and stop times for exposure identified in the exposure vidoes.

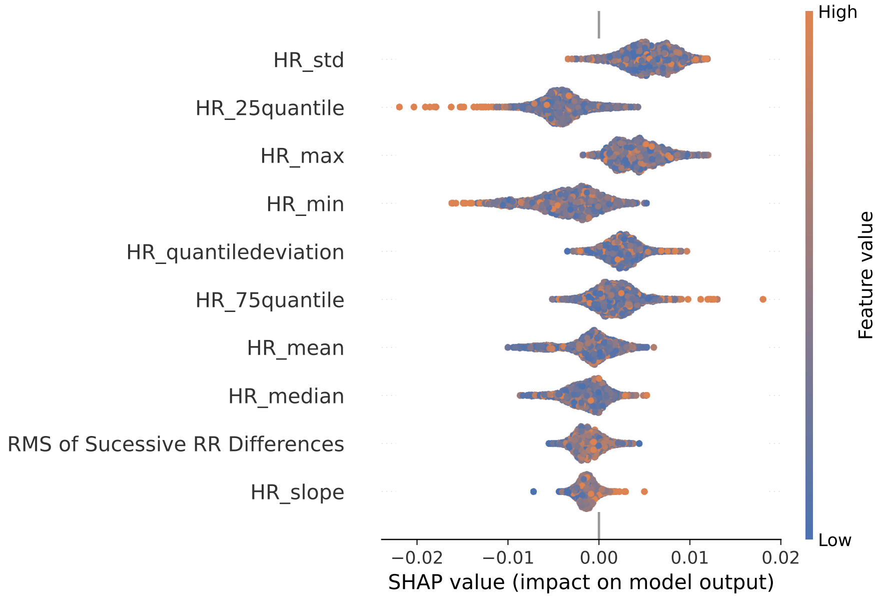


**Figure S7.** SHAP-values for features related to the heart rate (HR) and the root mean square (RSM) of RR differences using random cross-validation.

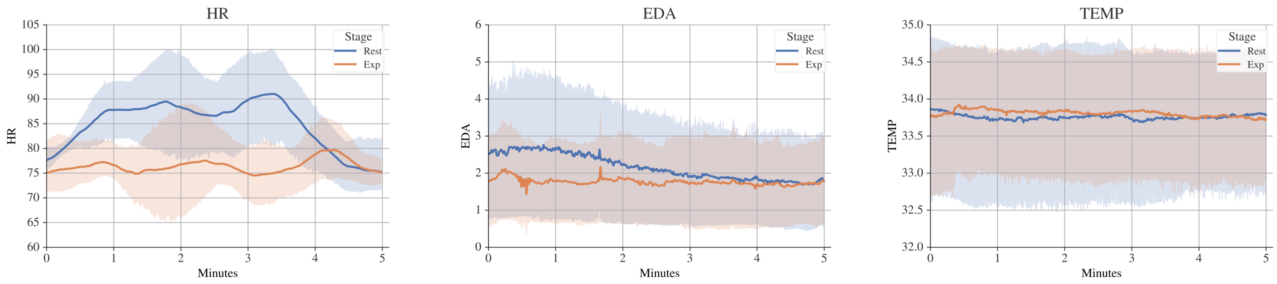


**Figure S8.** Mean heart rate (HR), electrodermal activity (EDA) and skin temperature for adolescents with OCD (n=5) under conditions of rest and exposure.

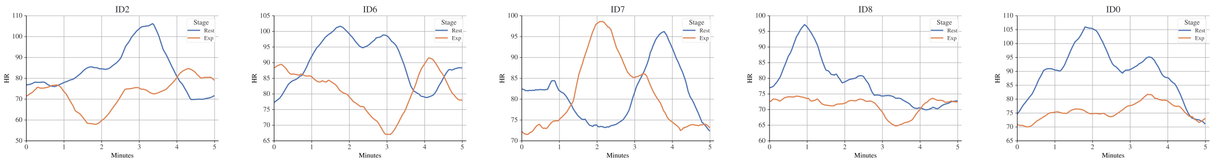


**Figure S9.** Heart rate (HR) during conditions of rest and exposure at Time 1 for five participants whose time stamps could be synchronized.

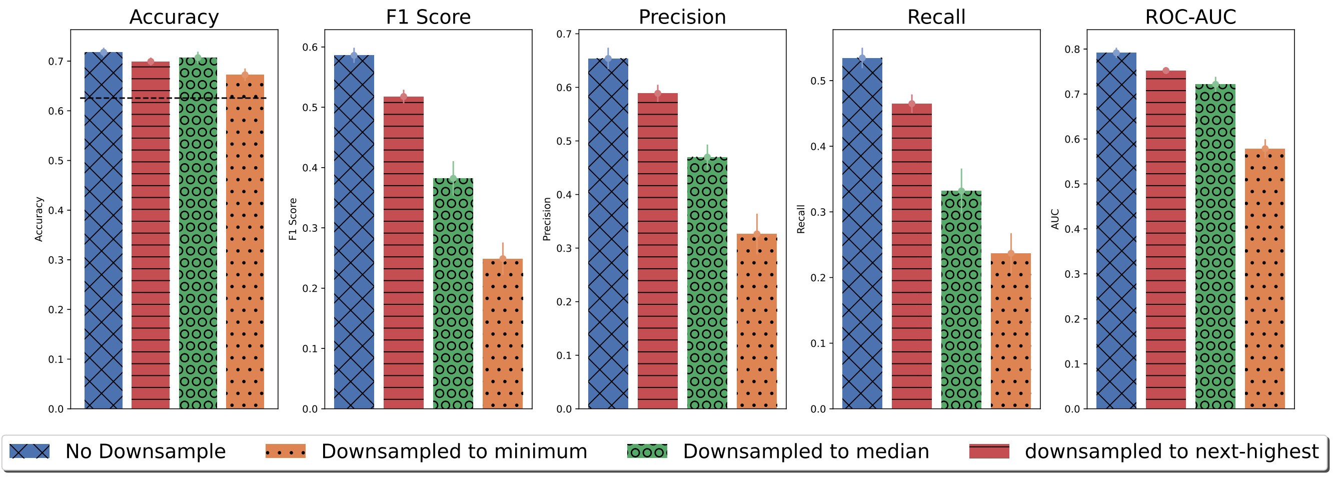


**Figure S10.** Performance comparison of two-layer random 10-fold cross-validation, in which data from participants with a large number of observations (OCD events and nonevents) have been randomly down-sampled to match the amount from participants with fewer observations. We provide p-values for the hypothesis of equal AUC between down-sampled models and models with no down-sampling (Hanley and McNeil, 1982). Down-sampling to participant with minimum observations, $< 10^{-4}$. Down-sampling to participant with median observations, $1.2 \cdot 10^{-4}$. Down-sampling to next highest, $7 \cdot 10^{-4}$. The dashed line represents the average value accuracy achieved by majority guessing.

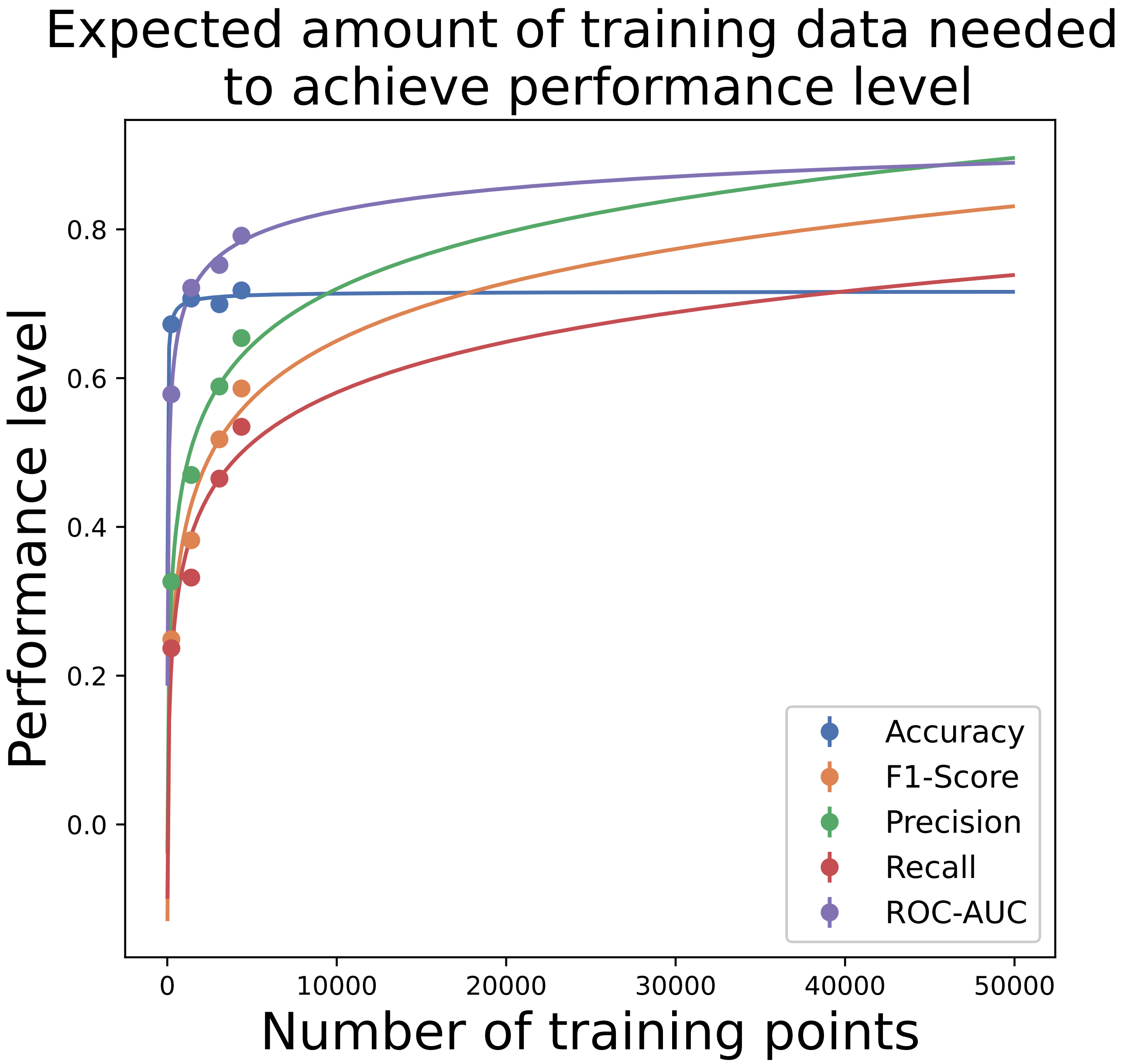


**Figure S11.** Estimated amount of training data required to achieve the given performance level in accuracy, F1-score, precision, recall, and ROC-AUC. Inverse power law fit to the performance levels obtained by downsampling high observation participants using two-layer 10-fold cross-validation Figueroa et al. (2012).

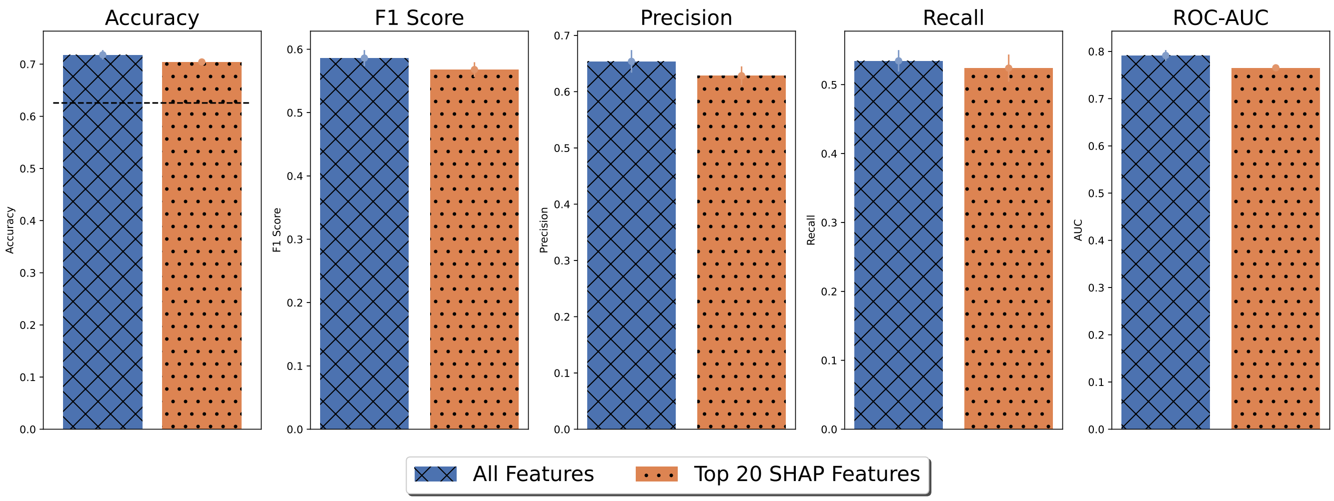


**Figure S12.** P-value for the hypothesis of equal AUC between top 20 SHAP features and all features: $7.4 \cdot 10^{-3}$.
